# Empirical Scoring Functions for Structure-Based Virtual Screening: Applications, Critical Aspects, and Challenges

**DOI:** 10.3389/fphar.2018.01089

**Published:** 2018-09-24

**Authors:** Isabella A. Guedes, Felipe S. S. Pereira, Laurent E. Dardenne

**Affiliations:** Grupo de Modelagem Molecular em Sistemas Biológicos, Laboratório Nacional de Computação Científica, Petrópolis, Brazil

**Keywords:** structure-based drug design, molecular docking, virtual screening, scoring function, binding affinity prediction, machine learning

## Abstract

Structure-based virtual screening (VS) is a widely used approach that employs the knowledge of the three-dimensional structure of the target of interest in the design of new lead compounds from large-scale molecular docking experiments. Through the prediction of the binding mode and affinity of a small molecule within the binding site of the target of interest, it is possible to understand important properties related to the binding process. Empirical scoring functions are widely used for pose and affinity prediction. Although pose prediction is performed with satisfactory accuracy, the correct prediction of binding affinity is still a challenging task and crucial for the success of structure-based VS experiments. There are several efforts in distinct fronts to develop even more sophisticated and accurate models for filtering and ranking large libraries of compounds. This paper will cover some recent successful applications and methodological advances, including strategies to explore the ligand entropy and solvent effects, training with sophisticated machine-learning techniques, and the use of quantum mechanics. Particular emphasis will be given to the discussion of critical aspects and further directions for the development of more accurate empirical scoring functions.

## Introduction

The drug discovery process required to enable a new compound to reach the market as an innovative therapeutic entity is significantly expensive and time-consuming (Mullard, 2014; DiMasi et al., 2016; Mignani et al., 2016). In this context, research groups and pharmaceutical industry have extensively included computer-aided drug design (CADD) approaches in their drug discovery pipeline to increase the potential of finding newer and safer drug candidates (Ban et al., 2017; Barril, 2017; Usha et al., 2017). Structure-based drug design (SBDD) methods, which require the three-dimensional structure of the macromolecular target, have been widely employed in successful campaigns (Bortolato et al., 2012; Danishuddin and Khan, 2015; Rognan, 2017). Although important challenges and some limitations have been addressed, many efforts have been made aiming the improvement of existing methods and the development of innovative approaches. Molecular docking is one of the most used SBDD approaches with several reviews published at the present time (Guedes et al., 2014; Ferreira et al., 2015; Yuriev et al., 2015; Pagadala et al., 2017; Dos Santos et al., 2018), and has been continuously explored by the scientific community to develop more sophisticated and accurate strategies. Docking aims to predict binding modes and affinity of a small molecule within the binding site of the receptor target of interest, supporting the researcher in the understanding of the main physicochemical features related to the binding process. Docking-based virtual screening (VS) consists of large-scale docking with a growing number of success cases reported (Villoutreix et al., 2009; Matter and Sotriffer, 2011; Rognan, 2017). Examples of docking programs are AutoDockVina (Trott and Olson, 2010), UCSF DOCK (Allen et al., 2015), GOLD (Jones et al., 1997), and Glide (Friesner et al., 2004, 2006a). Beyond the standalone software, web servers such as the DockThor Portal^1^ (de Magalhães et al., 2014), MTiOpenScreen^2^ (Labbé et al., 2015), HADDOCK^3^ (van Zundert et al., 2016), and DOCK Blaster^4^ (Irwin et al., 2009) provide to the scientific community friendly user interface and satisfactory time response of docking results.

The fast evaluation of docking poses generated by the search method and the accurate prediction of binding affinity of top-ranked poses is essential in VS protocols. In this context, scoring functions emerge as a straightforward and fast strategy despite limited accuracy, remaining as the main alternative to be applied in VS experiments (Huang et al., 2010). Moreover, the development of more accurate scoring functions is strategic in the field of SBDD and remains a challenging task, especially in the hit-to-lead optimization (Enyedy and Egan, 2008) and *de novo* design (Liu et al., 2017). Although there is no universal scoring function with significant reliability for all molecular systems, some important strategies were explored. Examples of free online resources for predicting protein-ligand binding affinities without the dependency a docking program are BAPPL server ^5^ (Jain and Jayaram, 2005) CSM-lig ^6^ (Pires and Ascher, 2016) and K_DEEP_^7^ (Jiménez Luna et al., 2018).

The development of an empirical scoring function requires three components (Pason and Sotriffer, 2016): (i) descriptors that describe the binding event, (ii) a dataset composed of three-dimensional structure of diverse protein–ligand complexes associated with the corresponding experimental affinity data, and (iii) a regression or classification algorithm to calibrate the model establishing a relationship between the descriptors and the experimental affinity. The empirical models differ in the number and type of descriptors; the algorithm adopted for training the model; and the number, the diversity, and the quality data of protein–ligand complexes used during the parameterization process.

According to the algorithm used for training, the scoring function can be linear (i.e., sum of weighted terms) or nonlinear (i.e., nonlinear relationship between the descriptors). It is important to highlight that even the multiple linear regression (MLR) algorithm, frequently used to calibrate linear scoring functions, is also a machine-learning technique. However, the term “machine-learning-based” scoring function is usually defined in the literature to refer to complex/nonlinear models developed using sophisticated machine-learning techniques to approximate nonlinear problems, such as random forests (RF), support-vector machines (SVM), and deep learning (DL) methods. The linear scoring functions are also referred as “classical” scoring functions. However, we will not adopt the “classical” nomenclature to avoid confusion with scoring functions based on classical force fields. In this work, we will adopt the nomenclature “linear” for the MLR scoring functions and “nonlinear” for models trained with more complex machine-learning techniques.

## Goals of Scoring Functions

During the docking process, the search algorithm investigates a vast amount of conformations for each molecule of the compound library. In this step, the scoring functions evaluate the quality of these docking poses, guiding the search methods toward relevant ligand conformations. The first requirement for a useful scoring function is to be able to distinguish the experimentally observed binding modes – associating them with the lowest binding energies of the energy landscape – from all the other poses found by the search algorithm (*pose prediction*). The second goal is to classify active and inactive compounds (VS), and the third is the prediction of the absolute binding affinity, ranking compounds correctly according to their potency (*binding affinity prediction*) (Jain and Nicholls, 2008; Cheng et al., 2009; Li et al., 2014c). The last one is the most challenging task, mainly in *de novo* design and lead optimization, since small differences in the compound could lead to drastic changes in binding affinity (Schneider and Fechner, 2005). An ideal scoring function would be able to perform the three tasks. However, given several limitations of current scoring functions, they exhibit different accuracies on distinct tasks due to modeling assumptions and simplifications made during their development phase, being intrinsically associated with the main purpose of the evaluated scoring function (Li et al., 2014b). In this context, docking protocols can adopt different scoring functions for each step, e.g., one can use a fast scoring function to predict binding modes and further predict affinities employing a more sophisticated scoring function specific for affinity prediction.

Current docking methods and the associated scoring functions exhibit good pose prediction power if one assumes an adequate preparation of the system and if the target flexibility does not play a significant role (Corbeil et al., 2012; Chaput and Mouawad, 2017). However, the detection of active compounds among a set of decoy compounds and the accurate prediction of binding affinity remain challenging tasks, even when induced fit and entropy effects are not important for binding (Gohlke and Klebe, 2002; Damm-Ganamet et al., 2013; Yuriev and Ramsland, 2013; Grinter and Zou, 2014; Smith et al., 2016). In VS experiments, it is mandatory the use of a scoring function capable of, at least, discriminating active from inactive molecules.

Scoring functions are typically divided into three main classes (Wang et al., 2003): *force field-based*, *knowledge-based*, and *empirical*. Liu and Wang (2015) recently proposed a new classification scheme, suggesting classifying current scoring functions as *physics-based*, *regression-based*, *potential of mean force*, and *descriptor-based*. Herein we will follow the traditional classification proposed by Wang et al. (2002) since we believe it is more general and is capable to classify adequately scoring functions according to the main development strategy adopted.

*Force field-based* functions consist of a sum of energy terms from a classical force field, usually considering the interaction energies of the protein–ligand complex (non-bonded terms) and the internal ligand energy (bonded and non-bonded terms), whereas the solvation energy can be computed by continuum solvation models such as the Poisson–Boltzmann (PB) or the related Generalized Born (GB) (Gilson et al., 1997; Zou and Kuntz, 1999). Examples of force field-based scoring functions include DOCK (Meng et al., 1992) and DockThor (de Magalhães et al., 2014).

*Knowledge-based* scoring functions are based on the statistical analysis of interacting atom pairs from protein–ligand complexes with available three-dimensional structures. These pairwise-atom data are converted into a pseudopotential, also known as a mean force potential, that describes the preferred geometries of the protein–ligand pairwise atoms. Examples include DrugScore (Velec et al., 2005) and PMF (Muegge, 2006).

*Empirical* scoring functions are developed to reproduce experimental affinity data (Pason and Sotriffer, 2016) based on the idea that it is possible to correlate the free energy of binding to a set of non-related variables. The coefficients associated with the functional terms are obtained through regression analysis using known binding affinity data of experimentally determined structures. LUDI was the first empirical scoring function developed in the pioneering work of Böhm (1992) for predicting the absolute binding free energy from atomic (3D) structures of protein–ligand complexes. Other examples of empirical scoring functions include ChemScore (Eldridge et al., 1997), ID-Score (Li et al., 2013), and GlideScore (Friesner et al., 2004, 2006a). Some empirical scoring functions (also referred as *hybrid* scoring functions) were developed using a mixture of force field-based, contact-based, and knowledge-based descriptors, such as DockTScore from the DockThor program (empirical and force-field based) (de Magalhães et al., 2014; Guedes et al., 2016), SMoG2016 (empirical and knowledge-based) (Debroise et al., 2017), and GalaxyDock BP2 Score (empirical, knowledge-based, and force-field based) (Baek et al., 2017).

The main focus of this review is the state-of-the-art concerning empirical scoring functions motivated by two main reasons. First, the methodology behind this type of scoring function could be fast enough to be used in large-scale structure-based VS and *de novo* design studies. Secondly, the use of modern sophisticated machine-learning techniques and the increasing availability of protein–ligand structures and measured binding affinity data could increase considerably the accuracy of empirical scoring functions to be useful in computer-aided SBDD experiments. In the following sections, we will discuss crucial aspects concerning their development, successful applications, limitations, and future perspectives.

## Descriptors of Empirical Scoring Functions

### Intermolecular Interactions

Empirical scoring functions have implemented specific terms accounting for intermolecular interactions, such as van der Waals and electrostatic potentials. For example, the Lennard-Jones potential describes the attractive forces (e.g., dispersion forces) and the intrinsic repulsive force between two separated atoms as a function of the interatomic distances (Jones, 1924a,b). Examples of empirical scoring functions using Lennard-Jones potentials are ID-Score (Li et al., 2013) and LISA (Zheng and Merz, 2011). X-Score (Wang et al., 2002) is an example of a scoring function that adopts a softened version of the Lennard-Jones potential instead of the conventional 12-6 potential.

Although all interatomic forces are of electrostatic or electromagnetic origin, the name “electrostatic” is conventionally used to describe forces between polar atoms and is usually represented by the Coulomb potential in both force field-based and empirical scoring functions. Glide (Friesner et al., 2006a) and DockThor (de Magalhães et al., 2014) are examples of scoring functions that implement the Coulomb potential for computing electrostatic interactions.

Some scoring functions include a specific term for hydrogen bonds interactions, commonly through two approaches: (i) by using specific force field-based parameters associated to the van der Waals and electrostatic energy potentials; (ii) by using a directional term, where the hydrogen bond contribution is a function of the deviation of the geometric parameters from those of an ideal hydrogen bond.

GlideScore employs the approach (i) to calculate hydrogen bonds between polar atom pairs, while the Glide XP Score applies the strategy (ii) to account for distinct categories of hydrogen bonds such as neutral–neutral, charged–charged, and neutral–charged interactions (Friesner et al., 2004, 2006b). The DockThor scoring function, which is based on the MMFF94S force field, has also implemented the strategy (i), reducing the size of the polar hydrogen atom when it is involved in hydrogen-bonding interactions (i.e., interacting with a hydrogen bond acceptor) (Halgren, 1996). X-Score adopts the approach (ii) and does not consider explicitly the hydrogen atoms, adopting a concept of “root” atom. In the LUDI implementation of the approach (ii), there are specific parameters for neutral hydrogen bonds and salt bridges (Böhm, 1994). However, some empirical functions do not differentiate hydrogen bonds between charged and neutral atom pairs, e.g., X-Score (Wang et al., 2002) and FlexX (Rarey et al., 1996). ID-Score is an example of a scoring function that uses both approaches: (i) to account for electrostatic interactions between charged groups and (ii) for hydrogen-bonding interactions (Li et al., 2013). The AutoDock4 scoring function employs a directional term based on a 10/12 potential (similar to the Lennard-Jones potential) dependent of the angle deviation from an ideal H-bond interaction with the protein. Besides the improvement in affinity predictions, the inclusion of a polar desolvation might be crucial to avoid overestimation of hydrogen bonds, since the H-bond formation is directly related with the desolvation of polar atoms.

Despite the importance in considering metal ions, it can be also a source of inaccuracy when using non-specific scoring functions, since the real contribution of interaction metal ions can be underestimated – in the case of simple counting of metal-atom interacting pairs – or overestimated – when using Coulomb potential with formal charges. For example, LUDI (Böhm, 1994), ChemScore (Eldridge et al., 1997), and SFCscore (Sotriffer et al., 2008) implement a contact-based term that attributes 1 to each pair metal–ligand atom within a distance criteria, and lower scores when the distance becomes larger than the specified criteria until an upper limit of distance, attributing the score 0 for larger distances. AutoDock4_Zn_ has implemented a specific force-field-based potential for the zinc ion to consider both geometric and energetic components of the metal–ligand interaction, achieving better performance for pose prediction in redocking experiments (Santos-Martins et al., 2014).

Many studies have highlighted the influence of halogen bonds (X-bonds) on enhancing binding affinity against several targets and the computational methods developed so far (Desiraju et al., 2013; Ford and Ho, 2016). Given the importance of this specific interaction in the hit and lead identification, some scoring functions have incorporated special treatment for X-bonds, such as XBScore (Zimmermann et al., 2015), ScorpionScore (Kuhn et al., 2011), and AutoDockVinaXB (Koebel et al., 2016).

### Desolvation

The desolvation contribution to the binding affinity arising from the formation of the protein–ligand complex with the release of water molecules to the bulk solvent can be separated into two distinct effects: the nonpolar and the polar desolvation. The nonpolar desolvation, favorable to binding, is related to the hydrophobic effect when transferring nonpolar molecular surface from the bulk water to a medium that is nonpolar, as is the case of many protein binding cavities (Tanford, 1980; Williams and Bardsley, 1999; Freire, 2008). At the same time, the desolvation of polar or charged groups of the protein or ligand is unfavorable to binding when the formed solute–solvent interactions are not effectively satisfied upon the protein–ligand binding (Blaber et al., 1993; Kar et al., 2013). In this context, many scoring functions have implemented desolvation terms to introduce the hydrophobic effect and/or penalize buried and not interacting polar/charged atoms after protein–ligand binding to improve binding affinity predictions.

The X-Score is a consensus scoring (CS) function based on three distinct strategies to represent the favorable contribution of the desolvation event related to the hydrophobic effect: hydrophobic surface (X-Score^HS^), hydrophobic matching (X-Score^HM^), and hydrophobic contact algorithms (X-Score^HC^) (Wang et al., 2002). The first one is the hydrophobic surface algorithm (X-Score^HS^), where the hydrophobic effect is proportional to the ligand hydrophobic surface in contact with the solvent accessible surface of the protein. The second is the hydrophobic matching algorithm (X-Score^HM^), the same algorithm adopted in the SCORE function (Wang et al., 1998) that calculates the hydrophobic contribution as a function of the logP of each ligand atom and the respective lipophilicity of surrounding protein atoms. The third and simplest method is the hydrophobic contact algorithm (X-Score^HC^), which approximates the hydrophobic effect through the contact between protein–ligand pairs of lipophilic atoms.

LUDI adopts an approach similar to the X-Score^HS^ (Böhm, 1994), while ChemScore (Eldridge et al., 1997) implements the algorithm similar to the X-Score^HC^. Fresno scoring function (Rognan et al., 1999) implements a more sophisticated method using the resolution of the linear form of the PB equation using finite difference methods. Cyscore (Cao and Li, 2014) considers the protein shape through a curvature-dependent surface-area term for hydrophobic free energy calculation, leading to a significant improvement on affinity prediction performance on PDBbind benchmarking sets.

The unfavorable desolvation effect from burying polar groups after ligand binding also plays an important role in the binding event, but it is commonly neglected by most scoring functions (Kar et al., 2013; Li et al., 2014c; Cramer et al., 2017). Some efforts have been made to implement specific penalization terms developed with distinct approaches to account for the polar desolvation, such as in the scoring functions ICM (Abagyan et al., 1994; Totrov and Abagyan, 1999; Fernández-Recio et al., 2004), XP GlideScore (Friesner et al., 2006a), LigScore (Krammer et al., 2005), and DockTScore (de Magalhães et al., 2014; Guedes et al., 2016).

The use of more sophisticated methods based on molecular dynamics (MD), such as MM-PBSA and MM-GBSA, have been used in conjunction with empirical scoring functions to predict binding affinities. MM-PBSA and the related MM-GBSA, considered as “end-point” approaches since all calculations are based on the initial and final states of the simulation, rely on MD simulations to compute the polar and nonpolar contributions of the protein–ligand binding event. A classical force field is utilized to compute the potential energy, and the solvation energy is calculated with an implicit solvation model. PB and GB are continuum electrostatic models used to calculate the electrostatic part of the solvation energy that treats the protein and the ligand as low-dielectric regions while considering the aqueous solvent as a high-dielectric medium (Honig et al., 1993). When associated with a surface-area-dependent term (SA), they lead to the implicit solvation models PB (PBSA) (Sitkoff et al., 1994) and Generalized Born (GBSA) (Still et al., 1990; Qiu et al., 1997). Sun et al. (2014) evaluated the performance of MM-PBSA and MM-GBSA methods using several protocols with 1864 protein–ligand complexes from PDBbind v2011 dataset. They concluded that although similar results were observed, MM-GBSA is less sensitive to the investigated systems and is more suitable to be used in general cases (e.g., reverse docking, which is widely used to predict the receptor target(s) of a compound). Inspired by the promising results obtained with GBSA, Zou and Kuntz (1999) implemented a GBSA scheme into the DOCK program as an alternative scoring function and obtained improved binding affinity predictions due to a better description of electrostatic and desolvation effects. More recently, Zhang X. et al. (2017) also obtained significant improvement on binding affinity prediction of antithrombin ligands when rescoring the top-scored docking poses from VinaLC docking engine with MM-GBSA. Spiliotopoulos et al. (2016) successfully integrated a damped version of MM-PBSA with the HADDOCK scoring function to predict binding poses and affinity of protein–peptide complexes.

### Ligand Entropy

Configurational entropy is related to the loss of flexibility of the ligand upon binding. It can be represented as a sum of the conformational (*S*_conf_) and the vibrational ($S_{\mathrm{vib}}^{o}$) entropies (Schäfer et al., 2002; Chang et al., 2007). In the energy landscape framework of the protein–ligand binding event, the former reflects the number of occupied energy wells and the last express the average width of the occupied wells. *S*_conf_ is related to the reduction of the number of ligand accessible conformations upon binding, while $S_{\mathrm{vib}}^{o}$ is mainly caused by the restriction of rotational amplitude inside the binding site when compared to the unbounded state (Chang et al., 2007; Gilson and Zhou, 2007).

Given the difficulty in modeling entropic effects for Δ*G*_bind_, scoring functions generally neglect their contributions or adopt simplified algorithms to approximate entropies in a straightforward manner (Jain, 2006). Scoring functions such as LUDI (Böhm, 1994) and X-Score (Wang et al., 2002) consider the entropic loss due to the restriction of rotational and translational degrees of freedom implicitly in the regression constant Δ*G*_0_. Surflex approximates such entropic loss as the logarithm of the ligand molecular weight multiplied by a scale factor related to the rough mass dependence of the translational and rotational entropies (Jain, 1996).

The restriction of the rotatable bonds of the ligand after the formation of the protein–ligand complex also promotes an entropic loss (*S*_conf_) that is unfavorable to the binding affinity. Some scoring functions have implemented specific terms in a rough approximation to account for entropic contributions of the ligand, as the most used strategies: (i) proportional to the number of rotatable bonds, and (ii) considering the environment of each rotatable bond, i.e., only penalize rotatable bonds that are in contact with the protein. LUDI (Böhm, 1994) and Fresno (Rognan et al., 1999) implement the approach (i) while ChemScore (Eldridge et al., 1997) and ID-Score (Li et al., 2013) use variations of the strategy (ii).

Inspired by the successful application of the energy landscape theory in protein folding and biomolecular binding (Jackson and Fersht, 1991; Miller and Dill, 1997; Baker, 2000), researchers make use of the multiple binding modes predicted by docking programs to describe the binding energy landscape. For example, Wei et al. (2010) developed two new parameters extracted from the multiple binding modes, generated by the AutoDock 3.05 program, and combined them for classification purposes using logistic regression to distinguish true binders among high-scored decoys. The new proposed scheme considered the energy gap (i.e., the difference between the binding energy of the native binding mode and the average binding energy of other binding modes – the *thermodynamic stability* of the native state) and the number of local binding wells (*kinetic accessibility*). This strategy was successfully applied in the neuraminidase and cyclooxygenase-2 systems from the DUD database, with even improved accuracy when associated with the docking scores. Grigoryan et al. (2012) also successfully applied the energy gap to distinguish true binders from decoys in several protein targets from DUD on single and multiple-receptor VS experiments, achieving superior performance than the ICM scoring function.

### Descriptors Based on the Counting of Atom Pairs

With the advance of sophisticated machine-learning algorithms, an increasing number of scoring functions based on a pool of simplistic descriptors have emerged, such as the counting of protein–ligand atom pairs and ligand-based properties. In the literature, such scoring functions are also known as “descriptor-based” or “machine-learning based.” It is important to note that this kind of scoring functions are also empirical models, since (i) the algorithms commonly used to derive the models, such as the classical MLR or the robust RF, are machine-learning methods^8^, (ii) the attributes used to describe the binding event are, in fact, descriptors, independently of their functional form, physical meaning, and complexity degree.

The success of descriptors based on the simple counting of atom pairs is associated with two important aspects: (i) amount and definition not limited by complex implementations or physical meaning assumptions, and (ii) practically eliminate the necessity of a detailed preparation of the structures, correct assignment of atom types, and physical quantities (e.g., atomic partial charges). Many papers in the recent literature describe outstanding results for binding affinity prediction and active/inactive classification using this more pragmatic approach (Ballester and Mitchell, 2010; Pereira et al., 2016; Wójcikowski et al., 2017). However, the conjunction of nonlinear models and more straightforward atom counting descriptors is subjected to significant criticisms (Gabel et al., 2014). Among the main critics we can highlight: (i) insensitiveness to the protonation state of the ligands and receptor residues; (ii) insensitiveness to the ligand pose; and (iii) facilitate the inclusion of methodological artifacts due to overtraining even when using large training sets.

## Training and Test Sets

### Datasets

The availability of protein–ligand structures with measured binding data has been increased due to efforts on data collection, such as PDBbind-CN (Liu et al., 2015, 2017), DUD-E (Mysinger et al., 2012), and DEKOIS (Bauer et al., 2013) projects.

PDBbind-CN is a source of biomolecular complexes with protein–ligand structure determined experimentally with the associated binding data manually collected from their original reference (Liu et al., 2015). The current release (version 2017) contains 17,900 structures (14,761 protein–ligand complexes) and is annually updated to keep up with the growth of the Protein Data Bank (Berman et al., 2000). The “refined set” is a subset composed of high-quality datasets constructed according to several criteria concerning the quality of the structures, the affinity data, and the nature of the complex, being considered one of the largest datasets of structures available for the development and validation of docking methodologies and scoring functions. Collected affinities comprise a large interval of values, ranging from 1.2 pM (1.2 × 10^−12^ M) to 10 mM (1.0 × 10^−3^ M). Also, PDBbind-CN provides a benchmarking named “core set” widely used for comparative assessment of scoring functions in predicting affinities (Li Y. et al., 2018). The core set is a subset of the refined set constructed using the following protocol: (i) firstly, protein structures with identity of sequence higher than 90% were grouped leading to 65 clusters associated with different protein families; (ii) only the clusters composed of at least five members were considered to construct the core set; and (iii) for each of these clusters, only the complexes with the lowest, the medium, and the highest affinities were selected to the final composition of the core set. A significant drawback of PDBbind-CN datasets is the insufficient information regarding negative data (i.e., experimentally confirmed inactive compounds).

The DUD-E dataset is an enhanced version of the original DUD set and has been widely used to train and validate scoring functions (Huang et al., 2006; Mysinger et al., 2012). It is composed of 102 targets with corresponding active, inactive, marginal, and decoy compounds. Although the number of ligands (i.e., active compounds) significantly varies for each target, a proportion of 50 decoys per ligand is kept for all 102 macromolecules. Decoys are presumed, not experimentally verified, to be inactive compounds since they are chosen to be topologically distinct from ligands but exhibiting similar physicochemical properties. The use of decoys instead of validated inactive compounds remains a major drawback for most datasets since no experimental activity are reported for them, and the number of confirmed inactive molecules is too scarce (Lagarde et al., 2015; Chaput et al., 2016b; Réau et al., 2018).

DEKOIS 2.0 is composed of 81 benchmarking sets for 80 protein targets of therapeutic relevance, including nonconventional targets such as protein–protein interaction complexes (Bauer et al., 2013). Active compounds and the associated binding affinity were retrieved from BindingDB applying several filters to remove pan assay interference (PAINS) compounds, weak binders, reactive groups, and undefined stereocenters. To derive a structurally diverse data set, for each protein target the active compounds were clustered into 40 groups according to the Tanimoto structural similarity and only the most potent compound of each cluster was selected. For each active molecule, 30 structurally diverse decoys molecules from ZINC database were selected according to an improved protocol to that used in the first version of DEKOIS dataset (Vogel et al., 2011), including the detection and removing of latent actives in the decoy set (LADS). Although DUD-E and DEKOIS 2.0 share a common structure of active and decoys compounds, they are complementary since there is a small overlap between them: only four protein targets present in DEKOIS 2.0 overlaps with the DUD-E dataset.

Scoring functions can be developed based on either experimental structures (i.e., protein–ligand structure experimentally determined) or conformations predicted with docking programs. The structure source (i.e., experimental or docked) is an important point to consider. The use of benchmarking sets such as DUD-E and DEKOIS2.0 is directly dependent on the docking program adopted since the experimental structures of the protein–ligand complexes are not available as in the PDBbind datasets. In fact, the scoring function training or validation in VS experiments using these datasets is performed with no warranty that the ligand poses were correctly predicted.

### Training, Validation, and Test Sets

The dataset is commonly separated into three subsets without overlapping structures: (i) the training set, (ii) the validation set, and (iii) the test set (also known as “external validation set”).

The *training* set is utilized to calibrate the parameters of the scoring function and to learn the rules that establish a quantitative relationship between the descriptors and the experimental affinity. The *validation* is used to assess the generalization error^9^ guiding the model tuning and selection. Once the best model is chosen, it is then applied to the *test* set to evaluate the real predictive capacity of the model.

There is a tradeoff between the size of the training and validation/test sets. Whereas the use of an extensive validation/test set is useful in providing a better estimate of the generalization error, this usually implicates in a smaller dataset to be utilized in the training phase (Abu-Mostafa et al., 2012). Studies evaluating the influence of the training size for the performance of linear and nonlinear scoring functions for affinity prediction demonstrated that MLR becomes insensitive to the growth of the training size whereas larger training sets can lead to an overall better accuracy of nonlinear scoring functions (Ding et al., 2013; Ain et al., 2015; Li et al., 2015a,b; Li H. et al., 2018).

In this context, cross-validation emerges as an alternative strategy to estimate the generalization error without strictly changing the training set size. Cross-validation experiments consist of continuously splitting the original training set of size *N* into two parts *K* times (*K*-fold cross-validation): a smaller set of size *V* for validation (*V* = *N*/*K*) and a larger set of the remaining *T* instances (*T* = *N−V*) for training (e.g., leave-one-out cross-validation considers *V* = 1). Different schemes of cross-validation have been adopted and explored to train linear and nonlinear models (Shao, 1993; Golbraikh and Tropsha, 2002; Kramer and Gedeck, 2010; Ballester and Mitchell, 2011; Wójcikowski et al., 2017). For example, in the recent work of Wójcikowski et al. (2017), they performed fivefold cross-validations using the DUD-E dataset. Three distinct splitting strategies were considered: *horizontal*, *vertical*, and *per-target*. In the *horizontal* split, all folds necessarily contain protein–ligand complexes from all protein targets (i.e., each protein target is present in both training and test sets). In the *vertical* split, the protein targets present in the test set do not have representative structures in the training set. This evaluation simulates those cases where the protein target of interest was not present during the training phase. Finally, in the *per-target* split, the training and test are performed for each protein target (i.e., 102 unique machine-learning models relative to the 102 DUD-E targets), simulating the construction and validation of target-specific scoring functions.

It is important to keep in mind that training, validation, and test sets must never have protein–ligand complexes in common at the same time. Furthermore, the test set must be composed of instances not used in the training process at any moment. Thus, the test set must be used only for evaluating the predictive performance of different scoring functions, and no decision should be taken based on the performance for this dataset to avoid useless comparisons due to artificially high correlations.

#### Benchmarking and Evaluation Metrics

Standard benchmarks are of great importance for an objective assessment of scoring functions providing a reproducible and reliable way to compare different methods. PDBbind (Liu et al., 2015), DUD-E (Mysinger et al., 2012), and DEKOIS 2.0 (Bauer et al., 2013) are examples of widely used benchmarks for evaluating scoring functions.

Many evaluation metrics are used to quantify the performance of scoring functions in pose prediction, active/inactive classification, and affinity prediction. A special issue on *Evaluation of Computational Methods* collects several high-quality papers covering the main aspects of the problem in evaluating and comparing distinct methodologies, highlighting the strengths and weakness of widely used metrics (Stouch, 2008). Recently, Huang and Wong (2016) developed an inexpensive method – the screening performance index (SPI) – to evaluate VS methods that correlate with BEDROC with less computational cost, since it discards the necessity of docking decoy compounds (i.e., only considers the docking of active molecules).

Scoring functions are generally evaluated regarding four aspects related to the three goals of scoring functions aforementioned (Liu et al., 2017):

*Docking power*: the ability of a scoring function in detecting the native binding mode from decoy poses as the top-ranked solution. The root-mean square deviation (RMSD) is the most commonly used metric to assess the docking power performance.

*Screening power*: the ability of a scoring function in correctly distinguishing active compounds from inactive molecules. The screening power test does not require that the scoring function correctly predict the absolute binding affinity. The screening power is usually quantified by BEDROC and enrichment factor (EF).

*Ranking power*: the ability of a scoring function in rank correctly the compounds according to the binding affinities against the *same* target protein. The Spearman correlation coefficient (*R*_S_) and Kendall’s tau are metrics widely used for assessing the ranking power of scoring functions.

*Scoring power*: the ability of a scoring function in rank correctly the compounds according to the binding affinities against *distinct* target proteins. It is important to note that the scoring power test considers the absolute value of the affinity prediction, requiring that the predicted and experimentally observed binding affinities have a linear correlation. This performance is widely assessed by the Pearson correlation coefficient (*R*_P_), and the root-mean squared error (RMSE).

The predictive performance of scoring functions may vary between different benchmarking experiments due to factors such as: (i) composition of the dataset, (ii) structural quality of the complexes, (iii) level of experience of the researches performing the experiments, and (iv) protocol of preparation of the complexes (Yuriev and Ramsland, 2013). Although ranking scoring functions according to their performances for affinity prediction on benchmark sets highlights the more competitive models, it is important to observe that small differences in the calculated performances are generally insufficient to state which scoring function performs better than other when comparing the top-ranked models. Since most benchmarking studies evaluate scoring functions on a few hundred complexes, small differences in Spearman correlation coefficient between 0.05 and 0.15, for example, lack statistical significance (Carlson, 2013, 2016). Thus, larger benchmarking sets composed of high-quality protein–ligand complexes structures are required for a reliable comparison of docking methodologies and scoring functions.

In addition to the well-known benchmarking sets, prospective evaluations are of substantial importance since the blinded predictions simulate real experiments of VS campaigns. Drug Design Data Resource (D3R^10^) periodically provide pharmaceutical-related benchmark datasets and a *Grand Challenge* as a blinded community challenge with unpublished data (Gathiaka et al., 2016). According to the results obtained in the *Grand Challenge 2*, it is clear that the pose prediction task is well performed for many methodologies, but scoring is still a very challenging task, even when the crystal structures are provided (Gaieb et al., 2018). Even with the crystal structures of 36 complexes at *Stage 2*, the maximum Kendall’s tau achieved was 0.46, reinforcing the great deal in correctly ranking a set of compounds. Performances and detailed description of the protocols adopted are provided at the D3R *Grand Challenge 2* website^11^ and on the scientific reports published on a special issue of Journal of Computer-Aided Molecular Design (Gaieb et al., 2018).

In the last version, D3R *Grand Challenge 3* (GC3), the participants had also to deal with even more challenging tasks, such as the selectivity identification for kinases, assessing the ability of the scoring functions in identifying large changes in affinity due to small structural changes in the ligand (*kinase activity cliff*), and the influence of kinase mutations on protein–ligand affinity (*kinase mutants*).

The broad profile of the D3R *Grand Challenges*, regarding chemical space diversity and affinity data carefully collected, makes their datasets one of the more reliable sources to evaluate docking and scoring methods, providing useful guidelines and best practices for further VS campaigns and methodological improvements.

### The Accuracy of Input Structural and Binding Data

Important issues regarding the quality of structural and affinity data must be considered for the development, validation, and application of scoring functions in VS experiments. Reliable protein–ligand structures usually comply these criteria: good resolution (2.5 Å or better), fully resolved electron density for the entire ligand and the surrounding binding-site residues, and without significant influences from crystal packing on the observed binding mode (Cole et al., 2011).

**FIGURE 1:**
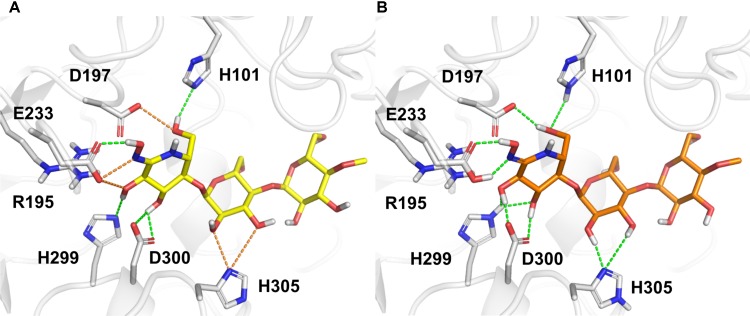
The structure of α-amylase complexed with the inhibitor MeG2-GHIL (PDB code 1U33) as **(A)** provided by PDBbind and **(B)** after manual preparation. Bad and favorable polar contacts are highlighted in orange and green dashes, respectively. D, aspartate; E, glutamate or glutamic acid; H, histidine; R, arginine.

The correct assignment of both protein and ligand protonation/tautomeric states with respect to the experimental pH, Asn/Gln/His flips, and defined stereocenters of the compounds are crucial, requiring a careful inspection of the structures (Kalliokoski et al., 2009; Martin, 2009; Petukh et al., 2013; Sastry et al., 2013). Indeed, the preparation of protein–ligand complexes has a direct influence on training and evaluation of scoring functions, mainly for scoring functions based on force-field descriptors. For example, the initial automatic preparation of the structures performed by PDBbind did not provide an optimized hydrogen bond network and appropriate assignment of protonation/tautomeric states of the α-amylase and MeG2-GHIL complex [**Figure 1**, PDB code 1U33; Numao et al., 2004]. The careful inspection and correction of such complexes comprise a time-consuming and challenging task, but they are particularly important when hydrogen atoms are considered explicitly. In such cases, the wrong orientation of hydrogen atoms can lead to high van der Waals energies, underestimation of hydrogen bond interactions, and incorrect electrostatic repulsions between charged/polar groups. Despite many efforts made for collecting even more extensive and better quality datasets, little attention has been paid to the careful preparation of the protein–ligand structures, usually relying on automatic procedures (Bauer et al., 2013). In this context, scoring functions mainly composed of simple contact-based descriptors (element–element pair counting) emerge to circumvent the complicated preparation required in large datasets for VS.

Especially for affinity prediction purposes, the use of datasets with curated affinity data is essential for reliable predictions and benchmarking. For example, the PDBbind refined set follows several criteria concerning the bioactivity manually collected from the original reference (Liu et al., 2015): (i) only complexes with known dissociation constants (*K*_d_) or inhibition constants (*K*_i_) are allowed, (ii) no complexes with extremely low (*K*_d_ or *K*_i_ > 10 mM) or extremely high (*K*_d_ or *K*_i_ < 1 pM) affinities are accepted, and (iii) estimated values are rejected, e.g., *K*_d_ ∼ 1 nM or *K*_i_ > 10 μM. Despite the efforts in collecting high-quality affinity data, many factors such as the inherent experimental error can be a source of inaccuracies, limiting the average prediction error achievable on large datasets (Shoichet, 2006; Ferreira et al., 2009; Sotriffer and Matter, 2011; Kramer et al., 2012). Furthermore, the use of decoys instead of confirmed inactive compounds has important impacts in training and measuring the performance of scoring functions (Chaput et al., 2016b; Réau et al., 2018).

## Machine Learning

### Regression and Classification

Scoring functions can be developed using *regression* methods to reproduce continuous (e.g., binding constants) or *classification* methods to reproduce binary affinity data (e.g., active/inactive). It is possible to use scoring functions trained with regression methods to classify active and inactive molecules given a predetermined range of affinity data for defining active and inactive compounds (Ain et al., 2015). It is also possible to use both classification and regression approaches to deal with the same problem of binding affinity prediction. For example, Pason and Sotriffer (2016) used a strategy of classifying the complexes using algorithms such as KNN and further generating linear regression models for each cluster achieving predictive performances comparable to that obtained by the nonlinear scoring function trained with RF. Many sophisticated machine-learning techniques automatically generate local models for similar training points (e.g., locally weighted regression), being able to classify the new instances automatically and use different regression models according to specific properties without explicitly defining classes based on such descriptors.

### Linear Versus Nonlinear Scoring Functions

Scoring functions can also be classified as “linear” and “nonlinear” models (Artemenko, 2008).

Linear regression is one of the simplest learning algorithms and is widely used as a starting point in the development of nonlinear regression models (Bishop, 2006). A linear empirical scoring function can be written as a sum of independent terms such as:

$$\Delta G_{\mathrm{binding}}=c_{0}+c_{1}\Delta G_{\mathrm{vdW}}+c_{2}\Delta G_{\mathrm{hbond}}+c_{3}\Delta G_{\mathrm{entropy}}$$

where *c_i_* is the weighting coefficients of the respective Δ*G*_i_ terms, adjusted to reproduce affinity data based on the training set. In the example, Δ*G*_vdW_ is a van der Waals potential, Δ*G*_hbond_ is a specific term accounting for hydrogen bonds, and Δ*G*_entropy_ is related to the ligand entropic loss upon binding.

The most crucial difference between linear and nonlinear scoring functions is that the former requires a predefined functional form (e.g., the sum of terms in the case of linear scoring functions), whereas the latter implicitly derives the mathematical relationship between the descriptors, allowing the combination of variables and higher order exponents for the terms. This advantage of nonlinear scoring functions partially circumvents the problematic modeling assumptions of linear models (Dill, 1997; Baum et al., 2010; Sotriffer, 2012).

Linear scoring functions developed to date have shown moderate correlations (*R*_P_ ∼ 0.6), whereas nonlinear models achieved significantly better correlations (*R*_P_ > 0.7) on benchmarking studies (Ashtawy and Mahapatra, 2012; Khamis and Gomaa, 2015; Wang and Zhang, 2017; Wójcikowski et al., 2017). RF, SVM, and more recently, DL, are nonlinear algorithms widely used to develop scoring functions.

The superiority of nonlinear models has also been confirmed through the rebuild of linear scoring functions using nonlinear algorithms, i.e., scoring functions trained with the same original descriptors of the correspondent linear model but with a different regression method. As an example, Zilian and Sotriffer (2013) trained a RF scoring function using the same SFCscore descriptors (named SFCscoreRF) and found a much improved model, with *R* = 0.779 significantly higher than those correlations obtained for the SFScore linear models (Pason and Sotriffer, 2016). Li et al. (2014a) investigated the replacement of MLR by RF for regression using the same Cyscore descriptors and found that the nonlinear model improved the affinity prediction. Furthermore, they also observed that larger training sets and describing the complexes with more descriptors have a positive impact in the predictive performance of the nonlinear models. Pason and Sotriffer (2016) demonstrated that it is possible to achieve similar high performances of nonlinear models through the development of a set of linear scoring functions trained using clustered – smaller and more homogeneous – datasets of protein–ligand complexes. In fact, many machine-learning techniques are based in this approach. For example, locally weighted linear regression automatically generate distinct “local” linear models weighting the training points according to their similarity with the instance to be predicted.

DL is considered as a promising approach to diverse drug discovery projects guided by the successes obtained in image and speech recognition problems (Zhang L. et al., 2017). Such methods take advantage of the recent increase in computational power and the ever-expanding availability of structural and binding data. DL methods are neural networks with many hidden layers, being capable to automatically learn the complicated relationship between the descriptors related to the protein–ligand binding. Recently, DL has been applied for pose/affinity prediction and active/inactive detection, exhibiting an outstanding performance when compared with several well-performing scoring functions developed with both linear and nonlinear approaches (Wallach et al., 2015; Khamis et al., 2016; Pereira et al., 2016; Ragoza et al., 2017; Jiménez Luna et al., 2018; Nguyen et al., 2018).

Despite nonlinear scoring functions have the main advantage of discarding the necessity of a pre-defined functional form, their main drawback is that they work as “black boxes” since the relationship between the descriptors is often vague, requiring careful use to avoid meaningless interpretations (Gabel et al., 2014). Together with the use of a significant amount of descriptors lacking physical meaning, nonlinear models offer the risk of producing excellent performance indexes due to overfitting and/or bias to the training set construction (e.g., capturing the rules adopted during the selection of active and decoy compounds) (Hawkins, 2004; Abu-Mostafa et al., 2012).

## Challenging Topics and Promising Strategies

### Protein Flexibility

Protein flexibility is still a great challenge for docking programs and scoring functions (Cavasotto and Singh, 2008; Tuffery and Derreumaux, 2012; Buonfiglio et al., 2015; Spyrakis and Cavasotto, 2015; Kurkcuoglu et al., 2018). Most docking methodologies adopt a single, rigid conformation of the receptor, due to the high computational cost and methodological limitations proportional to the increase in the degree of flexibility. However, over the last decades, many strategies have been implemented in docking programs to consider some degree of flexibility in the targeted, such as soft potentials and ensemble docking. In this context, the development of scoring functions adapted for flexible receptor docking is crucial to achieve real improvements in pose and affinity prediction (Totrov and Abagyan, 1997; Wei et al., 2002; Fischer et al., 2014; Ravindranath et al., 2015; Lam et al., 2017; Kong et al., 2018). Ferrari et al. (2004) implemented the fast and methodologically simple soft-docking strategy into the DOCK program, softening the repulsive term of the Lennard-Jones potential, allowing small overlaps between the protein and the ligand atoms. They also validated the methodology in VS studies of potential ligands of the T4 lysozyme and the aldol reductase and obtained better results than using regular docking strategies. Ensemble docking implicitly considers the receptor flexibility by docking the ligand on a set of protein conformations instead of a single conformation, being capable to simulate large-scale receptor flexibility (Korb et al., 2012). Recently, Fischer et al. (2014) successfully identified new ligands targeting specific receptor conformations of cytochrome c peroxidase using a flexible docking method that samples and weights protein conformations guided by experimentally derived conformations, integrating the Boltzmann-weighted energy penalties related with the protein flexibility to the DOCK3.7 scoring function. Despite the many efforts made to include the protein flexibility in VS experiments, the complex and multifactorial framework of flexible protein–ligand binding is still a great challenge (Bottegoni et al., 2011; Nunes-Alves and Arantes, 2014; Antunes et al., 2015; Buonfiglio et al., 2015; Kong et al., 2018). Whereas the high computational cost related with sampling protein conformations and docking large compound libraries can be overcome with the use of high-performance computing platforms, weighing such conformations and integrating them with the scoring functions remains a hindrance for accurate estimation of binding affinities on flexible systems.

### Solvation

Water molecules play an essential role in the ligand–protein binding process. Besides the hydrophobic and desolvation effects, individual water molecules can stabilize the ligand binding mode through the formation of water bridges or a water-mediated hydrogen-bond network (Poornima and Dean, 1995; Levy and Onuchic, 2006). The correct prediction of the free energy of binding associated to the ligand displacement of water molecules is a key challenge for the currently available docking scoring functions (Riniker et al., 2012; Spyrakis and Cavasotto, 2015; Bodnarchuk, 2016). An interesting approach is the use of a water-mapping protocol based on the post trajectory analysis of explicit solvent MD. This analysis is based on the inhomogeneous solvation theory and tries to predict the free energy cost of moving a water molecule from a protein hydration site into the bulk solvent (Yang et al., 2013). For instance, in the WScore docking methodology, the location and thermodynamics of explicit waters are predicted using WaterMap and integrated to the scoring function together with a desolvation term to penalize the associated desolvation of polar or uncharged groups of protein or ligand (Murphy et al., 2016). Many solvent mapping methods were evaluated on real drug design studies in a recent paper (Bucher et al., 2018), showing that solvent mapping methods could be important to help ligand optimization and to correctly rank compounds to assist synthetic prioritization. However, these approaches only calculate the solvent contribution to the free energy and must be combined with other methods to be used for lead optimization or VS.

Recently, Bodnarchuk (2016) published an extensive review of water-placement methods helpful for locating conserved water molecules within the protein binding site to be considered explicitly during the docking simulation. Once the water molecules are identified, some docking engines have implemented strategies to treat water molecules explicitly with adapted scoring functions. The GOLD program considers all-atom and flexible water model able to rotate around its three principal axes, and rewards water displacement in the GoldScore or ChemScore scoring functions according to a balance between the loss of rigid-body entropy and the change in the interaction energies on binding to the protein cavity (Verdonk et al., 2005). In AutoDock4, explicit water molecules of the first hydration shell as represented as uncharged spheres directly attached to the ligand, whereas a hydration force field accounting for the entropic and enthalpic contributions, automatically predicts their potential in mediating protein–ligand interactions (Forli and Olson, 2012).

### Covalent Docking

All the discussion made in this review assumes that we are dealing with non-covalent inhibitors. In such cases, the identification and development of computer-aided strategies to identify or improve lead compounds are based on the identification of non-covalent interactions (e.g., electrostatic, van der Waals, hydrophobic interactions) to improve potency or increase selectivity. However, there is a whole class of inhibitors that form a covalent bond with their enzyme/receptor target (De Cesco et al., 2017). Covalent inhibitors can further be divided into two different categories according to whether inhibition is reversible or irreversible (Tuley and Fast, 2018). The development of covalent-docking methodologies capable of dealing with such type of inhibition is very important due to the potential advantages associated with covalent inhibitors (De Cesco et al., 2017), including (i) sustained duration of action leading to less frequent dosing, (ii) increased ligand efficiency, (iii) ability to inhibit targets with shallow binding sites previously categorized as “undruggable,” and (iv) increased ability to overcome resistant mutations, among others. The development of non-covalent inhibitors in a drug-design study is usually guided by the optimization of the affinity or dissociation constants (i.e., *K*_i_, *K*_d_, IC_50_). However, dealing with covalent inhibition is even more complex, and in order to address the full potential of a covalent-inhibitor we need not only to measure their affinities but also kinetic binding parameters (e.g., residence time *t*_r_, the average time that a ligand remains bound in the binding site) (De Cesco et al., 2017; Trani et al., 2018). The development of docking methodologies to predict poses and binding affinities of ligands that bind covalently to the receptor is a challenging task. Due to the increasing interest in covalent drugs, many non-covalent docking programs have developed covalent versions and some new docking programs focused on covalent ligands have been developed (Kumalo et al., 2015; Awoonor-Williams et al., 2017; De Cesco et al., 2017). GOLD (Jones et al., 1997), Autodock4 (Bianco et al., 2016), CovalentDock (Ouyang et al., 2013), CovDock (Zhu et al., 2014), DOCKovalent (London et al., 2014), and DOCK-TITE (Scholz et al., 2015) are some examples of docking programs that developed specific methodologies to deal with covalent-docking. These methodologies were discussed in recent reviews addressing covalent-inhibitors and covalent docking (Kumalo et al., 2015; Awoonor-Williams et al., 2017; De Cesco et al., 2017). Some of these methods try to include the complexity of the covalent inhibition introducing modifications into their non-covalent scoring functions. For example, the introduction of a Morse potential to describe the energy associated with the bond formation (CovalentDock). Two critical aspects in the future development of covalent scoring functions are the capacity to predict the kinetics of ligand binding (e.g., residence times) and the intrinsic reactivity of electrophilic and nucleophilic pairs of atoms (De Cesco et al., 2017).

### Quantum Mechanics

The use of quantum mechanical methods can improve the description of protein–ligand interactions and, in principle, could provide a more accurate binding affinity (Raha and Merz, 2005; Chaskar et al., 2017; Crespo et al., 2017; Cavasotto et al., 2018). This is particularly true when dealing with systems where the molecular recognition involves bond formation, π-stacking, cation-π, halogen bonding (i.e., σ-hole bonding), and polarization and charge transfer effects (Christensen et al., 2016). These non-classical interactions/effects are beyond the limits of classical methods and represent a significant challenge to the development of scoring functions to be used in computational drug design experiments. In particular, metal ions interactions are essential when dealing with metalloproteins and, due to the large changes in the electronic structure under ligand binding, are also a great challenge. In the last 10 years, important advances were made in computing hardware (e.g., Graphics Processing Units – GPUs), in the development of quantum algorithms to compute molecular wave functions (Dixon and Merz, 1997; Birgin et al., 2013), the development of more reliable semi-empirical quantum methods (Christensen et al., 2016; Yilmazer and Korth, 2016), and development of new hybrid QM/MM methods (Chaskar et al., 2017; Melo et al., 2018). These advances were essential to overcome the bottleneck of the high computational cost and are allowing the increasing use of QM methods in the prediction of protein–ligand binding affinities (Crespo et al., 2017). Recent high-quality reviews cover applications of explicit QM calculations in lead identification and optimization (Adeniyi and Soliman, 2017; Crespo et al., 2017; Cavasotto et al., 2018), development of QM methods for ligand binding affinity calculations (Ryde and Söderhjelm, 2016), and development of semi-empirical QM methods for non-covalent interactions (Christensen et al., 2016; Yilmazer and Korth, 2016).

The results obtained using QM or hybrid QM/MM-based methods are very encouraging when compared to the standard scoring functions, principally when dealing with metalloproteins (Chaskar et al., 2017; Pecina et al., 2018). Wang et al. (2011) rebuild the AutoDock4 scoring function using ligand partial charges calculated with QM methods and protein charges from the Amber99SB instead of the Gasteiger method, improving both pose and affinity predictions. Moreover, the results from the 2016 D3R Grand Challenge indicate that the use of QM/MM scoring could be a powerful strategy (Gao et al., 2018). Yang et al. (2015) developed and introduced the quantum mechanics-based term XBScore^QM^ as a combination of van der Waals and electrostatic potentials to describe the X-bond interactions into the AutoDock4 scoring function. The new scoring function achieved good performances on both pose and affinity prediction when compared against 12 diverse scoring functions, and increase predictive capacity to deal with protein–ligand complexes with X-bond interactions. Nevertheless, it is important to note that it is not guaranteed that QM-based approaches will always outperform standard scoring functions (Crespo et al., 2017) and they still face the same problems associated with the correct estimation of the solvent and other entropic effects to the protein–ligand binding free energy.

### Consensus Scoring

The combination of different scoring functions on a scoring scheme (CS) is considered as a promising data fusion strategy to improve VS enrichment, pose, and affinity prediction (Charifson et al., 1999; Bissantz et al., 2000; Yang et al., 2005; Kaserer et al., 2015; Chaput et al., 2016a; Chaput and Mouawad, 2017; Ericksen et al., 2017). The CS strategy could overcome to some extent the limitations faced by the single-scoring approach, for example, the inconsistent performances across different protein targets and chemical classes (Moitessier et al., 2009). Moreover, CS is frequently used in some extent together with ensemble docking methodology, where different scores are predicted for different conformations of the protein target under investigation (Park et al., 2009, 2010; Paulsen and Anderson, 2009; Kelemen et al., 2016; Baumgartner and Evans, 2018; Li D.-D. et al., 2018).

Since the pioneering work of Charifson et al. (1999), many consensus strategies were developed and assessed on several target proteins, such as cyclooxygenases (Kaserer et al., 2015), and β-secretases (Liu et al., 2012). For instance, Kaserer et al. (2015) applied CS on prospective VS studies against cyclooxygenases 1 and 2 and found that the chance of a compound to be truly active increases when more tools predicted it as active. In the very interesting work of Wang and Wang (2001), they provided a theoretical basis for the effectiveness of CS on affinity prediction. They demonstrated that CS works due to a simple statistical reason related to the law of large numbers: the mean value found by repeated independent predictions tends toward the real and expected value.

Traditional CS approaches combine the predictions of the scoring functions using statistical methods (e.g., arithmetic mean) or voting schemes (i.e., a vote replaces the absolute score predicted by each scoring function) (Terp et al., 2001; Wang and Wang, 2001; Wang et al., 2002; Bar-Haim et al., 2009; Ericksen et al., 2017). Nonlinear CS models were also developed to improve pose prediction and ranking compounds in VS experiments (Betzi et al., 2006; Teramoto and Fukunishi, 2007; Ashtawy and Mahapatra, 2015; Ericksen et al., 2017). For example, Ericksen et al. (2017) developed machine-learning CS using discrete mixture models and gradient boosting to combine the scores from eight docking programs and obtained improved performances than individual scoring functions on 21 targets from DUD-E dataset. In addition, they compared their machine-learning-based CS with individual scoring functions and traditional CS schemed, confirming that CS excel individual scoring functions performances in docking-based VS, being less sensitive to protein target variation.

### Tailored Scoring Functions for Protein Targets and Classes

Significant improvements in docking and VS accuracies are reported when employing target-specific scoring functions rather than non-specific models, using as training datasets protein–ligand complexes comprising specific molecular targets instead of a general dataset. Hence, it is expected that they could be more efficient in accounting for specific interactions and particular binding characteristics associated with a target class of interest (Seifert, 2009).

For instance, Logean et al. (2001) adapted the Fresno empirical scoring function to the class I MHC HLA-B^∗^2705 protein with a significant improvement in affinity prediction over six different traditional scoring functions. The GOLD program also implements a modified version of the ChemScore function, with an additional term that accounts for weak hydrogen bonds that claimed to be relevant for some kinase inhibitor binding (Pierce et al., 2002; Verdonk et al., 2004). The HADDOCK_PPI_ is a linear scoring function specifically developed to predict binding affinities of inhibitors of protein–protein interactions (iPPIs), which interact in uncommon binding cavities characterized by higher hydrophobicity, aromaticity, and molecular weight compared to enzyme inhibitors, as usually interacting within flatter, larger, and more hydrophobic binding sites than the enzyme catalytic sites (Morelli et al., 2011; Kuenemann et al., 2014). In a more recent work, a scoring function specific to Heat Shock Protein 90 (HSP90) was successfully designed and applied in VS (Santos-Martins, 2016). In general, nonlinear scoring functions specific for protein classes/targets also achieved superior performance than the generic models (Wang et al., 2015; Ashtawy and Mahapatra, 2018). Still, in the recent work of Wójcikowski et al. (2017), the target-specific scoring functions trained with RF only performed slightly better than generic models, with two-third of them increasing the EF_1%_ less than 10%. As an intriguing result, they found that tailored scoring functions are more beneficial for the protein targets with less active compounds than the others containing more actives, where the target-specific scoring functions exhibit similar performances to the generic model.

Despite encouraging results obtained for target-specific scoring functions, it is important to highlight that the requirement of a large training set to derive a robust scoring function might become a significant hindrance and source of inaccuracy. To overcome the lack of a sufficient amount of experimental structures, protein–ligand conformations used for training target-specific scoring functions are commonly obtained from docking experiments.

## Conclusion

The development of accurate empirical scoring functions to predict protein–ligand binding affinities is a key aspect in SBDD. In recent years, the increasing availability of protein–ligand structures with measured binding affinities and data sets containing active, decoy, and true inactive compounds are boosting the use of sophisticated machine-learning techniques to obtain better performing scoring functions. In the coming years, it is expected that the combination of larger training datasets, non-physical/simplified descriptors, and DL techniques will be a very promising research line to improve scoring functions for structure-based VS. Methodological advances will be dependent to the size and quality of the available datasets for training and benchmarking, and great care will be necessary to avoid artificial performances due to the increased capacity of these nonlinear methods to capture bias present in the training data. In this sense, blinded community challenges with unpublished data (e.g., D3R challenge) are essential to address the real performance of scoring functions and docking protocols. Looking to the other side of the methodological spectrum, it is exciting to note that the advance in computing power, the development of new algorithms to introduce protein flexibility and solvation/desolvation effects, and more reliable semi-empirical quantum methods are enabling the development and use of new methodological advances for challenging tasks, such as QM/MM-based methods and entropy estimation.

The full potential of scoring functions will be achieved when models accurate enough to be useful in hit-to-lead optimization and *de novo* design studies are developed. To reach this goal, a scoring function must be sensitive to the docking pose, *right for the right reasons* (Kolb and Irwin, 2009). Reliable predictions of ligand binding affinity remain a big challenge, but we expect that in the next years important advances associated to distinct methodological approaches will be achieved and, probably, will be combined into more effective computer-based drug design protocols.

## Author Contributions

IG and LD designed, wrote, and edited this review. FP contributed to designing and writing the review.

## Conflict of Interest Statement

The authors declare that the research was conducted in the absence of any commercial or financial relationships that could be construed as a potential conflict of interest.

## Footnotes

1 http://www.dockthor.lncc.br

2 http://bioserv.rpbs.univ-paris-diderot.fr/services/MTiOpenScreen/

3 http://haddock.science.uu.nl/services/HADDOCK2.2

4 http://blaster.docking.org/

5 www.scfbio-iitd.res.in/software/drugdesign/bappl.jsp

6 structure.bioc.cam.ac.uk/csm_lig

7 playmolecule.org/Kdeep

8 Indeed, according to the IUPAC Recommendations 2015, the term “machine learning” refers to *a computer algorithm that generate empirical models, (...), that is derived from the analysis of a training set for which all the necessary data are available* (Martin et al., 2016).

9 Generalization error is the expected error when the scoring function is evaluated on a dataset composed of new protein–ligand complexes (i.e., structures not used in the training step).

10 http://www.drugdesigndata.org

11 https://drugdesigndata.org//about/grand-challenge-2-evaluation-results

## References

[B1] Abagyan R. Totrov M. Kuznetsov D. (1994). ICM: a new method for protein modeling and design: applications to docking and structure prediction from the distorted native conformation. *J. Comput. Chem.* 15 488–506. 10.1002/jcc.540150503

[B2] Abu-Mostafa Y. S. Magdon-Ismail M. Lin H.-T. (2012). *Learning From Data.* United States: AMLBook.

[B3] Adeniyi A. A. Soliman M. E. S. (2017). Implementing QM in docking calculations: is it a waste of computational time? *Drug Discov. Today* 22 1216–1223. 10.1016/j.drudis.2017.06.012 28689054

[B4] Ain Q. U. Aleksandrova A. Roessler F. D. Ballester P. J. (2015). Machine-learning scoring functions to improve structure-based binding affinity prediction and virtual screening: machine-learning SFs to improve structure-based binding affinity prediction and virtual screening. *Wiley Interdiscip. Rev. Comput. Mol. Sci.* 5 405–424. 10.1002/wcms.1225 27110292 PMC4832270

[B5] Allen W. J. Balius T. E. Mukherjee S. Brozell S. R. Moustakas D. T. Lang P. T. (2015). DOCK 6: impact of new features and current docking performance. *J. Comput. Chem.* 36 1132–1156. 10.1002/jcc.23905 25914306 PMC4469538

[B6] Antunes D. A. Devaurs D. Kavraki L. E. (2015). Understanding the challenges of protein flexibility in drug design. *Exp. Opin. Drug Discov.* 10 1301–1313. 10.1517/17460441.2015.1094458 26414598

[B7] Artemenko N. (2008). Distance dependent scoring function for describing protein-ligand intermolecular interactions. *J. Chem. Inform. Model.* 48 569–574. 10.1021/ci700224e 18290639

[B8] Ashtawy H. M. Mahapatra N. R. (2012). A comparative assessment of ranking accuracies of conventional and machine-learning-based scoring functions for protein-ligand binding affinity prediction. *IEEEACM Trans. Comput. Biol. Bioinforma. IEEE ACM* 9 1301–1313. 10.1109/TCBB.2012.36 22411892

[B9] Ashtawy H. M. Mahapatra N. R. (2015). BgN-Score and BsN-Score: bagging and boosting based ensemble neural networks scoring functions for accurate binding affinity prediction of protein-ligand complexes. *BMC Bioinformatics* 16(Suppl. 4):S8. 10.1186/1471-2105-16-S4-S8 25734685 PMC4347622

[B10] Ashtawy H. M. Mahapatra N. R. (2018). Task-specific scoring functions for predicting ligand binding poses and affinity and for screening enrichment. *J. Chem. Inform. Model.* 58 119–133. 10.1021/acs.jcim.7b00309 29190087

[B11] Awoonor-Williams E. Walsh A. G. Rowley C. N. (2017). Modeling covalent-modifier drugs. *Biochim. Biophys. Acta BBA – Proteins Proteom.* 1865 1664–1675. 10.1016/j.bbapap.2017.05.009 28528876

[B12] Baek M. Shin W.-H. Chung H. W. Seok C. (2017). GalaxyDock BP2 score: a hybrid scoring function for accurate protein–ligand docking. *J. Comput. Aided Mol. Des.* 31 653–666. 10.1007/s10822-017-0030-9 28623486

[B13] Baker D. (2000). A surprising simplicity to protein folding. *Nature* 405 39–42. 10.1038/35011000 10811210

[B14] Ballester P. J. Mitchell J. B. O. (2010). A machine learning approach to predicting protein-ligand binding affinity with applications to molecular docking. *Bioinformatics* 26 1169–1175. 10.1093/bioinformatics/btq112 20236947 PMC3524828

[B15] Ballester P. J. Mitchell J. B. O. (2011). Comments on “leave-cluster-out cross-validation is appropriate for scoring functions derived from diverse protein data sets”: significance for the validation of scoring functions. *J. Chem. Inform. Model.* 51 1739–1741. 10.1021/ci200057e 21591735

[B16] Ban F. Dalal K. Li H. LeBlanc E. Rennie P. S. Cherkasov A. (2017). Best practices of computer-aided drug discovery: lessons learned from the development of a preclinical candidate for prostate cancer with a new mechanism of action. *J. Chem. Inform. Model.* 57 1018–1028. 10.1021/acs.jcim.7b00137 28441481

[B17] Bar-Haim S. Aharon A. Ben-Moshe T. Marantz Y. Senderowitz H. (2009). SeleX-CS: a new consensus scoring algorithm for hit discovery and lead optimization. *J. Chem. Inform. Model.* 49 623–633. 10.1021/ci800335j 19231809

[B18] Barril X. (2017). Computer-aided drug design: time to play with novel chemical matter. *Expert Opin. Drug Discov.* 12 977–980. 10.1080/17460441.2017.1362386 28756685

[B19] Bauer M. R. Ibrahim T. M. Vogel S. M. Boeckler F. M. (2013). Evaluation and optimization of virtual screening workflows with DEKOIS 2.0 *–* A public library of challenging docking benchmark sets. *J. Chem. Inform. Model.* 53 1447–1462. 10.1021/ci400115b 23705874

[B20] Baum B. Muley L. Smolinski M. Heine A. Hangauer D. Klebe G. (2010). Non-additivity of functional group contributions in protein-ligand binding: a comprehensive study by crystallography and isothermal titration calorimetry. *J. Mol. Biol.* 397 1042–1054. 10.1016/j.jmb.2010.02.007 20156458

[B21] Baumgartner M. P. Evans D. A. (2018). Lessons learned in induced fit docking and metadynamics in the drug design data resource grand challenge 2. *J. Comput. Aided Mol. Des.* 32 45–58. 10.1007/s10822-017-0081-y 29127581

[B22] Berman H. M. Westbrook J. Feng Z. Gilliland G. Bhat T. N. Weissig H. (2000). The protein data bank. *Nucleic Acids Res.* 28 235–242. 10592235 10.1093/nar/28.1.235 PMC102472

[B23] Betzi S. Suhre K. Chétrit B. Guerlesquin F. Morelli X. (2006). GFscore: a general nonlinear consensus scoring function for high-throughput docking. *J. Chem. Inform. Model.* 46 1704–1712. 10.1021/ci0600758 16859302

[B24] Bianco G. Forli S. Goodsell D. S. Olson A. J. (2016). Covalent docking using autodock: two-point attractor and flexible side chain methods. *Protein Sci. Publ. Protein Soc.* 25 295–301. 10.1002/pro.2733 26103917 PMC4815316

[B25] Birgin E. G. Martıìnez J. M. Martıìnez L. Rocha G. B. (2013). Sparse projected-gradient method as a linear-scaling low-memory alternative to diagonalization in self-consistent field electronic structure calculations. *J. Chem. Theory Comput.* 9 1043–1051. 10.1021/ct3009683 26588747

[B26] Bishop C. M. (2006). *Pattern Recognition and Machine Learning.* Switzerland: Springer

[B27] Bissantz C. Folkers G. Rognan D. (2000). Protein-based virtual screening of chemical databases. 1, evaluation of different docking/scoring combinations. *J. Med. Chem.* 43 4759–4767. 11123984 10.1021/jm001044l

[B28] Blaber M. Lindstrom J. D. Gassner N. Xu J. Heinz D. W. Matthews B. W. (1993). Energetic cost and structural consequences of burying a hydroxyl group within the core of a protein determined from Ala.f*wdarw*, ser and Val.fwdarw. Thr substitutions in T4 lysozyme. *Biochemistry* (Mosc.) 32 11363–11373. 10.1021/bi00093a013 8218201

[B29] Bodnarchuk M. S. (2016). Water, water, everywhere… It’s time to stop and think. *Drug Discov. Today* 21 1139–1146. 10.1016/j.drudis.2016.05.009 27210724

[B30] Böhm H. J. (1992). The computer program LUDI: a new method for the de novo design of enzyme inhibitors. *J. Comput. Aided Mol. Des.* 6 61–78. 1583540 10.1007/BF00124387

[B31] Böhm H. J. (1994). The development of a simple empirical scoring function to estimate the binding constant for a protein-ligand complex of known three-dimensional structure. *J. Comput. Aided Mol. Des.* 8 243–256. 7964925 10.1007/BF00126743

[B32] Bortolato A. Perruccio F. Moro S. (2012). “successful applications of in silico approaches for lead/drug discovery,” in *In-Silico Lead Discovery*, ed. Miteva M. A. (Emirate of Sharjah: Bentham Science Publishers), 163–175.

[B33] Bottegoni G. Rocchia W. Rueda M. Abagyan R. Cavalli A. (2011). Systematic exploitation of multiple receptor conformations for virtual ligand screening. *PLoS One* 6:e18845. 10.1371/journal.pone.0018845 21625529 PMC3098722

[B34] Bucher D. Stouten P. Triballeau N. (2018). Shedding light on important waters for drug design: simulations versus grid-based methods. *J. Chem. Inform. Model.* 58 692–699. 10.1021/acs.jcim.7b00642 29489352

[B35] Buonfiglio R. Recanatini M. Masetti M. (2015). Protein flexibility in drug discovery: from theory to computation. *ChemMedChem* 10 1141–1148. 10.1002/cmdc.201500086 25891095

[B36] Cao Y. Li L. (2014). Improved protein-ligand binding affinity prediction by using a curvature-dependent surface-area model. *Bioinform. Oxf. Engl.* 30 1674–1680. 10.1093/bioinformatics/btu104 24563257

[B37] Carlson H. A. (2013). Check your confidence: size really does matter. *J. Chem. Inform. Model.* 53 1837–1841. 10.1021/ci4004249 23909878 PMC3821705

[B38] Carlson H. A. (2016). Lessons learned over four benchmark exercises from the community structure-activity resource. *J. Chem. Inform. Model.* 56 951–954. 10.1021/acs.jcim.6b00182 27345761 PMC5217176

[B39] Cavasotto C. Singh N. (2008). Docking and high throughput docking: successes and the challenge of protein flexibility. *Curr. Comput. Aided-Drug Des.* 4 221–234. 10.2174/157340908785747474

[B40] Cavasotto C. N. Adler N. S. Aucar M. G. (2018). Quantum chemical approaches in structure-based virtual screening and lead optimization. *Front. Chem.* 6:188. 10.3389/fchem.2018.00188 29896472 PMC5986912

[B41] Chang C. A. Chen W. Gilson M. K. (2007). Ligand configurational entropy and protein binding. *Proc. Natl. Acad. Sci. U.S.A.* 104 1534–1539. 10.1073/pnas.0610494104 17242351 PMC1780070

[B42] Chaput L. Martinez-Sanz J. Quiniou E. Rigolet P. Saettel N. Mouawad L. (2016a). vSDC: a method to improve early recognition in virtual screening when limited experimental resources are available. *J. Cheminformatics* 8:1. 10.1186/s13321-016-0112-z 26807156 PMC4722699

[B43] Chaput L. Martinez-Sanz J. Saettel N. Mouawad L. (2016b). Benchmark of four popular virtual screening programs: construction of the active/decoy dataset remains a major determinant of measured performance. *J. Cheminformatics* 8:56. 10.1186/s13321-016-0167-x 27803745 PMC5066283

[B44] Chaput L. Mouawad L. (2017). Efficient conformational sampling and weak scoring in docking programs? Strategy of the wisdom of crowds. *J. Cheminformatics* 9:37. 10.1186/s13321-017-0227-x 29086077 PMC5468358

[B45] Charifson P. S. Corkery J. J. Murcko M. A. Walters W. P. (1999). Consensus scoring: a method for obtaining improved hit rates from docking databases of three-dimensional structures into proteins. *J. Med. Chem.* 42 5100–5109. 10.1021/jm990352k 10602695

[B46] Chaskar P. Zoete V. Röhrig U. F. (2017). On-the-fly QM/MM docking with attracting cavities. *J. Chem. Inform. Model.* 57 73–84. 10.1021/acs.jcim.6b00406 27983849

[B47] Cheng T. Li X. Li Y. Liu Z. Wang R. (2009). Comparative assessment of scoring functions on a diverse test set. *J. Chem. Inform. Model.* 49 1079–1093. 10.1021/ci9000053 19358517

[B48] Christensen A. S. Kubaø T. Cui Q. Elstner M. (2016). Semiempirical quantum mechanical methods for noncovalent interactions for chemical and biochemical applications. *Chem. Rev.* 116 5301–5337. 10.1021/acs.chemrev.5b00584 27074247 PMC4867870

[B49] Cole J. C. Korb O. Olsson T. S. G. Liebeschuetz J. (2011). “The basis for target-based virtual screening: protein structures,” in *Methods and Principles in Medicinal Chemistry*, ed. Sotriffer C. (Weinheim: Wiley-VCH Verlag GmbH & Co. KGaA), 87–114. 10.1002/9783527633326.ch4

[B50] Corbeil C. R. Williams C. I. Labute P. (2012). Variability in docking success rates due to dataset preparation. *J. Comput. Aided Mol. Des.* 26 775–786. 10.1007/s10822-012-9570-1 22566074 PMC3397132

[B51] Cramer J. Krimmer S. G. Heine A. Klebe G. (2017). Paying the Price of desolvation in solvent-exposed protein pockets: impact of distal solubilizing groups on affinity and binding thermodynamics in a series of thermolysin inhibitors. *J. Med. Chem.* 60 5791–5799. 10.1021/acs.jmedchem.7b00490 28590130

[B52] Crespo A. Rodriguez-Granillo A. Lim V. T. (2017). Quantum-mechanics methodologies in drug discovery: applications of docking and scoring in lead optimization. *Curr. Top. Med. Chem.* 17 2663–2680. 10.2174/1568026617666170707120609 28685695

[B53] Damm-Ganamet K. L. Smith R. D. Dunbar J. B. Stuckey J. A. Carlson H. A. (2013). CSAR benchmark exercise 2011–2012: evaluation of results from docking and relative ranking of blinded congeneric series. *J. Chem. Inform. Model.* 53 1853–1870. 10.1021/ci400025f 23548044 PMC3753884

[B54] Danishuddin M. Khan A. U. (2015). Structure based virtual screening to discover putative drug candidates: necessary considerations and successful case studies. *Methods* 71 135–145. 10.1016/j.ymeth.2014.10.019 25448480

[B55] De Cesco S. Kurian J. Dufresne C. Mittermaier A. K. Moitessier N. (2017). Covalent inhibitors design and discovery. *Eur. J. Med. Chem.* 138 96–114. 10.1016/j.ejmech.2017.06.019 28651155

[B56] de Magalhães C. S. Almeida D. M. Barbosa H. J. C. Dardenne L. E. (2014). A dynamic niching genetic algorithm strategy for docking highly flexible ligands. *Inform. Sci.* 289 206–224. 10.1016/j.ins.2014.08.002

[B57] Debroise T. Shakhnovich E. I. Chéron N. (2017). A hybrid knowledge-based and empirical scoring function for protein–ligand interaction: SMoG2016. *J. Chem. Inform. Model.* 57 584–593. 10.1021/acs.jcim.6b00610 28191941

[B58] Desiraju G. R. Ho P. S. Kloo L. Legon A. C. Marquardt R. Metrangolo P. (2013). Definition of the halogen bond (IUPAC Recommendations 2013). *Pure Appl. Chem.* 85 1711–1713. 10.1351/PAC-REC-12-05-10

[B59] Dill K. A. (1997). Additivity principles in biochemistry. *J. Biol. Chem.* 272 701–704. 10.1074/jbc.272.2.701 8995351

[B60] DiMasi J. A. Grabowski H. G. Hansen R. W. (2016). Innovation in the pharmaceutical industry: new estimates of R&D costs. *J. Health Econ.* 47 20–33. 10.1016/j.jhealeco.2016.01.012 26928437

[B61] Ding B. Wang J. Li N. Wang W. (2013). Characterization of small molecule binding. I. Accurate Identification of Strong Inhibitors in Virtual Screening. *J. Chem. Inform. Model.* 53 114–122. 10.1021/ci300508m 23259763 PMC3584174

[B62] Dixon S. L. Merz K. M. (1997). Fast, accurate semiempirical molecular orbital calculations for macromolecules. *J. Chem. Phys.* 107 879–893. 10.1063/1.474386

[B63] Dos Santos R. N. Ferreira L. G. Andricopulo A. D. (2018). Practices in molecular docking and structure-based virtual screening. *Methods Mol. Biol. Clifton NJ* 1762 31–50. 10.1007/978-1-4939-7756-7_3 29594766

[B64] Eldridge M. D. Murray C. W. Auton T. R. Paolini G. V. Mee R. P. (1997). Empirical scoring functions: I. The development of a fast empirical scoring function to estimate the binding affinity of ligands in receptor complexes. *J. Comput. Aided Mol. Des.* 11 425–445. 9385547 10.1023/a:1007996124545

[B65] Enyedy I. J. Egan W. J. (2008). Can we use docking and scoring for hit-to-lead optimization? *J. Comput. Aided Mol. Des.* 22 161–168. 10.1007/s10822-007-9165-4 18183356

[B66] Ericksen S. S. Wu H. Zhang H. Michael L. A. Newton M. A. Hoffmann F. M. (2017). Machine learning consensus scoring improves performance across targets in structure-based virtual screening. *J. Chem. Inform. Model.* 57 1579–1590. 10.1021/acs.jcim.7b00153 28654262 PMC5872818

[B67] Fernández-Recio J. Totrov M. Abagyan R. (2004). Identification of protein-protein interaction sites from docking energy landscapes. *J. Mol. Biol.* 335 843–865. 14687579 10.1016/j.jmb.2003.10.069

[B68] Ferrari A. M. Wei B. Q. Costantino L. Shoichet B. K. (2004). Soft docking and multiple receptor conformations in virtual screening. *J. Med. Chem.* 47 5076–5084. 10.1021/jm049756p 15456251 PMC1413506

[B69] Ferreira L. dos Santos R. Oliva G. Andricopulo A. (2015). Molecular docking and structure-based drug design strategies. *Molecules* 20 13384–13421. 10.3390/molecules200713384 26205061 PMC6332083

[B70] Ferreira R. S. Bryant C. Ang K. K. H. McKerrow J. H. Shoichet B. K. Renslo A. R. (2009). Divergent modes of enzyme inhibition in a homologous structure-activity series. *J. Med. Chem.* 52 5005–5008. 10.1021/jm9009229 19637873 PMC3760508

[B71] Fischer M. Coleman R. G. Fraser J. S. Shoichet B. K. (2014). Incorporation of protein flexibility and conformational energy penalties in docking screens to improve ligand discovery. *Nat. Chem.* 6 575–583. 10.1038/nchem.1954 24950326 PMC4144196

[B72] Ford M. C. Ho P. S. (2016). Computational tools to model halogen bonds in medicinal chemistry. *J. Med. Chem.* 59 1655–1670. 10.1021/acs.jmedchem.5b00997 26465079

[B73] Forli S. Olson A. J. (2012). A force field with discrete displaceable waters and desolvation entropy for hydrated ligand docking. *J. Med. Chem.* 55 623–638. 10.1021/jm2005145 22148468 PMC3319101

[B74] Freire E. (2008). Do enthalpy and entropy distinguish first in class from best in class? *Drug Discov. Today* 13 869–874. 10.1016/j.drudis.2008.07.005 18703160 PMC2581116

[B75] Friesner R. A. Banks J. L. Murphy R. B. Halgren T. A. Klicic J. J. Mainz D. T. (2004). Glide: a new approach for rapid, accurate docking and scoring. 1. method and assessment of docking accuracy. *J. Med. Chem.* 47 1739–1749. 10.1021/jm0306430 15027865

[B76] Friesner R. A. Murphy R. B. Repasky M. P. Frye L. L. Greenwood J. R. Halgren T. A. (2006a). Extra precision glide: docking and scoring incorporating a model of hydrophobic enclosure for protein-ligand complexes. *J. Med. Chem.* 49 6177–6196. 10.1021/jm051256o 17034125

[B77] Friesner R. A. Murphy R. B. Repasky M. P. Frye L. L. Greenwood J. R. Halgren T. A. (2006b). Extra precision glide: docking and scoring incorporating a model of hydrophobic enclosure for protein-ligand complexes. *J. Med. Chem.* 49 6177–6196. 10.1021/jm051256o 17034125

[B78] Gabel J. Desaphy J. Rognan D. (2014). Beware of machine learning-based scoring functions-on the danger of developing black boxes. *J. Chem. Inform. Model.* 54 2807–2815. 10.1021/ci500406k 25207678

[B79] Gaieb Z. Liu S. Gathiaka S. Chiu M. Yang H. Shao C. (2018). D3R grand challenge 2: blind prediction of protein–ligand poses, affinity rankings, and relative binding free energies. *J. Comput. Aided Mol. Des.* 32 1–20. 10.1007/s10822-017-0088-4 29204945 PMC5767524

[B80] Gao Y.-D. Hu Y. Crespo A. Wang D. Armacost K. A. Fells J. I. (2018). Workflows and performances in the ranking prediction of 2016 D3R Grand Challenge 2: lessons learned from a collaborative effort. *J. Comput. Aided Mol. Des.* 32 129–142. 10.1007/s10822-017-0072-z 28986733

[B81] Gathiaka S. Liu S. Chiu M. Yang H. Stuckey J. A. Kang Y. N. (2016). D3R grand challenge 2015: evaluation of protein-ligand pose and affinity predictions. *J. Comput. Aided Mol. Des.* 30 651–668. 10.1007/s10822-016-9946-8 27696240 PMC5562487

[B82] Gilson M. K. Given J. A. Head M. S. (1997). A new class of models for computing receptor-ligand binding affinities. *Chem. Biol.* 4 87–92. 9190290 10.1016/s1074-5521(97)90251-9

[B83] Gilson M. K. Zhou H.-X. (2007). Calculation of protein-ligand binding affinities. *Annu. Rev. Biophys. Biomol. Struct.* 36 21–42. 10.1146/annurev.biophys.36.040306.132550 17201676

[B84] Gohlke H. Klebe G. (2002). Approaches to the description and prediction of the binding affinity of small-molecule ligands to macromolecular receptors. *Angew. Chem. Int. Ed.* 41 2644–2676. 12203463 10.1002/1521-3773(20020802)41:15<2644::AID-ANIE2644>3.0.CO;2-O

[B85] Golbraikh A. Tropsha A. (2002). Beware of q2! J. *Mol. Graph. Model.* 20 269–276. 10.1016/s1093-3263(01)00123-1 11858635

[B86] Grigoryan A. V. Wang H. Cardozo T. J. (2012). Can the Energy gap in the protein-ligand binding energy landscape be used as a descriptor in virtual ligand screening? *PLoS One* 7:e46532. 10.1371/journal.pone.0046532 23071584 PMC3468575

[B87] Grinter S. Z. Zou X. (2014). Challenges, applications, and recent advances of protein-ligand docking in structure-based drug design. *Mol. Basel Switz.* 19 10150–10176. 10.3390/molecules190710150 25019558 PMC6270832

[B88] Guedes I. A. Barreto A. M. S. Miteva M. A. Dardenne L. E. (2016). Development of empirical scoring functions for predicting protein-ligand binding affinity. *Soc. Bras. Bioquim. Biol. Mol.* 1–174.

[B89] Guedes I. A. de Magalhães C. S. Dardenne L. E. (2014). Receptor–ligand molecular docking. *Biophys. Rev.* 6 75–87. 10.1007/s12551-013-0130-2 28509958 PMC5425711

[B90] Halgren T. A. (1996). Merck molecular force field. II. MMFF94 van der Waals and electrostatic parameters for intermolecular interactions. *J. Comput. Chem.* 17 520–552.

[B91] Hawkins D. M. (2004). The problem of overfitting. *J. Chem. Inform. Comput. Sci.* 44 1–12. 10.1021/ci0342472 14741005

[B92] Honig B. Sharp K. Yang A. S. (1993). Macroscopic models of aqueous solutions: biological and chemical applications. *J. Phys. Chem.* 97 1101–1109. 10.1021/j100108a002

[B93] Huang N. Shoichet B. K. Irwin J. J. (2006). Benchmarking sets for molecular docking. *J. Med. Chem.* 49 6789–6801. 10.1021/jm0608356 17154509 PMC3383317

[B94] Huang S.-Y. Grinter S. Z. Zou X. (2010). Scoring functions and their evaluation methods for protein-ligand docking: recent advances and future directions. *Phys. Chem. Chem. Phys.* 12 12899–12908. 10.1039/c0cp00151a 20730182 PMC11103779

[B95] Huang Z. Wong C. F. (2016). Inexpensive method for selecting receptor structures for virtual screening. *J. Chem. Inform. Model.* 56 21–34. 10.1021/acs.jcim.5b00299 26651874

[B96] Irwin J. J. Shoichet B. K. Mysinger M. M. Huang N. Colizzi F. Wassam P. (2009). Automated docking screens: a feasibility study. *J. Med. Chem.* 52 5712–5720. 10.1021/jm9006966 19719084 PMC2745826

[B97] Jackson S. E. Fersht A. R. (1991). Folding of chymotrypsin inhibitor 2. 1, Evidence for a two-state transition. *Biochemistry (Mosc.)* 30 10428–10435. 10.1021/bi00107a010 1931967

[B98] Jain A. N. (1996). Scoring noncovalent protein-ligand interactions: a continuous differentiable function tuned to compute binding affinities. *J. Comput. Aided Mol. Des.* 10 427–440. 8951652 10.1007/BF00124474

[B99] Jain A. N. (2006). Scoring functions for protein-ligand docking. *Curr. Protein Pept. Sci.* 7 407–420. 17073693 10.2174/138920306778559395

[B100] Jain A. N. Nicholls A. (2008). Recommendations for evaluation of computational methods. *J. Comput. Aided Mol. Des.* 22 133–139. 10.1007/s10822-008-9196-5 18338228 PMC2311385

[B101] Jain T. Jayaram B. (2005). An all atom energy based computational protocol for predicting binding affinities of protein-ligand complexes. *FEBS Lett.* 579 6659–6666. 10.1016/j.febslet.2005.10.031 16307743

[B102] Jiménez Luna J. Skalic M. Martinez-Rosell G. De Fabritiis G. (2018). KDEEP: Protein-ligand absolute binding affinity prediction via 3D-convolutional neural networks. *J. Chem. Inform. Model.* 58 287–296. 10.1021/acs.jcim.7b00650 29309725

[B103] Jones G. Willett P. Glen R. C. Leach A. R. Taylor R. (1997). Development and validation of a genetic algorithm for flexible docking. *J. Mol. Biol.* 267 727–748. 10.1006/jmbi.1996.0897 9126849

[B104] Jones J. E. (1924a). On the determination of molecular fields, I. From the variation of the viscosity of a gas with temperature. *Proc. R. Soc. Lond. Math. Phys. Eng. Sci.* 106 441–462. 10.1098/rspa.1924.0081

[B105] Jones J. E. (1924b). On the determination of molecular fields, II. From the equation of state of a gas. *Proc. R. Soc. Lond. Math. Phys. Eng. Sci.* 106 463–477. 10.1098/rspa.1924.0082

[B106] Kalliokoski T. Salo H. S. Lahtela-Kakkonen M. Poso A. (2009). The effect of ligand-based tautomer and protomer prediction on structure-based virtual screening. *J. Chem. Inform. Model.* 49 2742–2748. 10.1021/ci900364w 19928753

[B107] Kar P. Lipowsky R. Knecht V. (2013). Importance of polar solvation and configurational entropy for design of antiretroviral drugs targeting HIV-1 protease. *J. Phys. Chem. B* 117 5793–5805. 10.1021/jp3085292 23614718

[B108] Kaserer T. Temml V. Kutil Z. Vanek T. Landa P. Schuster D. (2015). Prospective performance evaluation of selected common virtual screening tools, case study: cyclooxygenase (COX) 1 and 2. *Eur. J. Med. Chem.* 96 445–457. 10.1016/j.ejmech.2015.04.017 25916906 PMC4444576

[B109] Kelemen Á. A. Kiss R. Ferenczy G. G. Kovács L. Flachner B. Lõrincz Z. (2016). Structure-based consensus scoring scheme for selecting class A aminergic GPCR fragments. *J. Chem. Inform. Model.* 56 412–422. 10.1021/acs.jcim.5b00598 26760056

[B110] Khamis M. Gomaa W. Galal B. (2016). Deep learning is competing random forest in computational docking. arXiv:1608.06665 [Preprint].

[B111] Khamis M. A. Gomaa W. (2015). Comparative assessment of machine-learning scoring functions on PDBbind 2013. *Eng. Appl. Artif. Intell.* 45 136–151. 10.1016/j.engappai.2015.06.021

[B112] Koebel M. R. Schmadeke G. Posner R. G. Sirimulla S. (2016). AutoDock VinaXB: implementation of XBSF, new empirical halogen bond scoring function, into AutoDock Vina. *J. Cheminform.* 8:27. 10.1186/s13321-016-0139-1 27195023 PMC4870740

[B113] Kolb P. Irwin J. J. (2009). Docking screens: right for the right reasons? *Curr. Top. Med. Chem.* 9 755–770. 19754393 10.2174/156802609789207091 PMC3383315

[B114] Kong X. Sun H. Pan P. Zhu F. Chang S. Xu L. (2018). Importance of protein flexibility in molecular recognition: a case study on Type-I1/2 inhibitors of ALK. *Phys. Chem. Chem. Phys.* 20 4851–4863. 10.1039/C7CP08241J 29383359

[B115] Korb O. Olsson T. S. G. Bowden S. J. Hall R. J. Verdonk M. L. Liebeschuetz J. W. (2012). Potential and limitations of ensemble docking. *J. Chem. Inform. Model.* 52 1262–1274. 10.1021/ci2005934 22482774

[B116] Kramer C. Gedeck P. (2010). Leave-cluster-out cross-validation is appropriate for scoring functions derived from diverse protein data sets. *J. Chem. Inform. Model.* 50 1961–1969. 10.1021/ci100264e 20936880

[B117] Kramer C. Kalliokoski T. Gedeck P. Vulpetti A. (2012). The experimental uncertainty of heterogeneous public ki data. *J. Med. Chem.* 55 5165–5173. 10.1021/jm300131x 22643060

[B118] Krammer A. Kirchhoff P. D. Jiang X. Venkatachalam C. M. Waldman M. (2005). LigScore: a novel scoring function for predicting binding affinities. *J. Mol. Graph. Model.* 23 395–407. 10.1016/j.jmgm.2004.11.007 15781182

[B119] Kuenemann M. A. Bourbon L. M. L. Labbé C. M. Villoutreix B. O. Sperandio O. (2014). Which three-dimensional characteristics make efficient inhibitors of protein–protein interactions? *J. Chem. Inform. Model.* 54 3067–3079. 10.1021/ci500487q 25285479

[B120] Kuhn B. Fuchs J. E. Reutlinger M. Stahl M. Taylor N. R. (2011). Rationalizing tight ligand binding through cooperative interaction networks. *J. Chem. Inform. Model.* 51 3180–3198. 10.1021/ci200319e 22087588 PMC3246350

[B121] Kumalo H. M. Bhakat S. Soliman M. E. S. (2015). Theory and applications of covalent docking in drug discovery: merits and pitfalls. *Mol. Basel Switz.* 20 1984–2000. 10.3390/molecules20021984 25633330 PMC6272664

[B122] Kurkcuoglu Z. Koukos P. I. Citro N. Trellet M. E. Rodrigues J. P. G. L. M. Moreira I. S. (2018). Performance of HADDOCK and a simple contact-based protein-ligand binding affinity predictor in the D3R grand challenge 2. *J. Comput. Aided Mol. Des.* 32 175–185. 10.1007/s10822-017-0049-y 28831657 PMC5767195

[B123] Labbé C. M. Rey J. Lagorce D. Vavruša M. Becot J. Sperandio O. (2015). MTiOpenScreen: a web server for structure-based virtual screening. *Nucleic Acids Res.* 43 W448–W454. 10.1093/nar/gkv306 25855812 PMC4489289

[B124] Lagarde N. Zagury J.-F. Montes M. (2015). Benchmarking data sets for the evaluation of virtual ligand screening methods: review and perspectives. *J. Chem. Inform. Model.* 55 1297–1307. 10.1021/acs.jcim.5b00090 26038804

[B125] Lam P. C.-H. Abagyan R. Totrov M. (2017). Ligand-biased ensemble receptor docking (LigBEnD): a hybrid ligand/receptor structure-based approach. *J. Comput. Aided Mol. Des.* 32 187–198. 10.1007/s10822-017-0058-x 28887659 PMC5767200

[B126] Levy Y. Onuchic J. N. (2006). Water mediation in protein folding and molecular recognition. *Annu. Rev. Biophys. Biomol. Struct.* 35 389–415. 10.1146/annurev.biophys.35.040405.102134 16689642

[B127] Li D.-D. Meng X.-F. Wang Q. Yu P. Zhao L.-G. Zhang Z.-P. (2018). Consensus scoring model for the molecular docking study of mTOR kinase inhibitor. *J. Mol. Graph. Model.* 79 81–87. 10.1016/j.jmgm.2017.11.003 29154212

[B128] Li H. Peng J. Leung Y. Leung K.-S. Wong M.-H. Lu G. (2018). The impact of protein structure and sequence similarity on the accuracy of machine-learning scoring functions for binding affinity prediction. *Biomolecules* 8:12. 10.3390/biom8010012 29538331 PMC5871981

[B129] Li Y. Su M. Liu Z. Li J. Liu J. Han L. (2018). Assessing protein-ligand interaction scoring functions with the CASF-2013 benchmark. *Nat. Protoc.* 13 666–680. 10.1038/nprot.2017.114 29517771

[B130] Li G.-B. Yang L.-L. Wang W.-J. Li L.-L. Yang S.-Y. (2013). ID-score: a new empirical scoring function based on a comprehensive set of descriptors related to protein–ligand interactions. *J. Chem. Inform. Model.* 53 592–600. 10.1021/ci300493w 23394072

[B131] Li H. Leung K.-S. Wong M.-H. Ballester P. (2015a). Low-quality structural and interaction data improves binding affinity prediction via random forest. *Molecules* 20 10947–10962. 10.3390/molecules200610947 26076113 PMC6272292

[B132] Li H. Leung K.-S. Wong M.-H. Ballester P. J. (2015b). Improving autodock vina using random forest: the growing accuracy of binding affinity prediction by the effective exploitation of larger data sets. *Mol. Inform.* 34 115–126. 10.1002/minf.201400132 27490034

[B133] Li H. Leung K.-S. Wong M.-H. Ballester P. J. (2014a). Substituting random forest for multiple linear regression improves binding affinity prediction of scoring functions: cyscore as a case study. *BMC Bioinformatics* 15:291. 10.1186/1471-2105-15-291 25159129 PMC4153907

[B134] Li H. Leung K.-S. Wong M.-H. Ballester P. J. (2014b). “The impact of docking pose generation error on the prediction of binding affinity,” in *Computational Intelligence Methods for Bioinformatics and Biostatistics Lecture Notes in Computer Science*, eds Serio C. D. Liò P. Nonis A. Tagliaferri R. (Berlin: Springer International Publishing), 231–241. 10.1007/978-3-319-24462-4_20

[B135] Li Y. Han L. Liu Z. Wang R. (2014c). Comparative assessment of scoring functions on an updated benchmark: 2, evaluation methods and general results. *J. Chem. Inform. Model.* 54 1717–1736. 10.1021/ci500081m 24708446

[B136] Liu J. Wang R. (2015). Classification of current scoring functions. *J. Chem. Inform. Model.* 55 475–482. 10.1021/ci500731a 25647463

[B137] Liu S. Fu R. Zhou L.-H. Chen S.-P. (2012). Application of consensus scoring and principal component analysis for virtual screening against β-secretase (BACE-1). *PLoS One* 7:e38086. 10.1371/journal.pone.0038086 22701601 PMC3372491

[B138] Liu Z. Li Y. Han L. Li J. Liu J. Zhao Z. (2015). PDB-wide collection of binding data: current status of the PDBbind database. *Bioinformatics* 31 405–412. 10.1093/bioinformatics/btu626 25301850

[B139] Liu Z. Su M. Han L. Liu J. Yang Q. Li Y. (2017). Forging the basis for developing protein-ligand interaction scoring functions. *Acc. Chem. Res.* 50 302–309. 10.1021/acs.accounts.6b00491 28182403

[B140] Logean A. Sette A. Rognan D. (2001). Customized versus universal scoring functions: application to class I MHC-peptide binding free energy predictions. *Bioorg. Med. Chem. Lett.* 11 675–679. 11266167 10.1016/s0960-894x(01)00021-x

[B141] London N. Miller R. M. Krishnan S. Uchida K. Irwin J. J. Eidam O. (2014). Covalent docking of large libraries for the discovery of chemical probes. *Nat. Chem. Biol.* 10 1066–1072. 10.1038/nchembio.1666 25344815 PMC4232467

[B142] Martin Y. C. (2009). Let’s not forget tautomers. *J. Comput. Aided Mol. Des.* 23 693–704. 10.1007/s10822-009-9303-2 19842045 PMC2776169

[B143] Martin Y. C. Abagyan R. Ferenczy G. G. Gillet V. J. Oprea T. I. Ulander J. (2016). Glossary of terms used in computational drug design, part II (IUPAC Recommendations 2015). *Pure Appl. Chem.* 88 239–264. 10.1515/pac-2012-1204

[B144] Matter H. Sotriffer C. (2011). “Applications and success stories in virtual screening,” in *Methods and Principles in Medicinal Chemistry*, ed. Sotriffer C. (Weinheim: Wiley-VCH Verlag GmbH & Co. KGaA), 319–358.

[B145] Melo M. C. R. Bernardi R. C. Rudack T. Scheurer M. Riplinger C. Phillips J. C. (2018). NAMD goes quantum: an integrative suite for hybrid simulations. *Nat. Methods* 15 351–354. 10.1038/nmeth.4638 29578535 PMC6095686

[B146] Meng E. C. Shoichet B. K. Kuntz I. D. (1992). Automated docking with grid-based energy evaluation. *J. Comput. Chem.* 13 505–524. 10.1002/jcc.540130412

[B147] Mignani S. Huber S. Tomás H. Rodrigues J. Majoral J.-P. (2016). Why and how have drug discovery strategies in pharma changed? What are the new mindsets? *Drug Discov. Today* 21 239–249. 10.1016/j.drudis.2015.09.007 26376356

[B148] Miller D. W. Dill K. A. (1997). Ligand binding to proteins: the binding landscape model. *Protein Sci.* 6 2166–2179. 10.1002/pro.5560061011 9336839 PMC2143563

[B149] Moitessier N. Englebienne P. Lee D. Lawandi J. Corbeil C. R. (2009). Towards the development of universal, fast and highly accurate docking/scoring methods: a long way to go: docking/scoring methods-a review. *Br. J. Pharmacol.* 153 S7–S26. 10.1038/sj.bjp.0707515 18037925 PMC2268060

[B150] Morelli X. Bourgeas R. Roche P. (2011). Chemical and structural lessons from recent successes in protein-protein interaction inhibition (2P2I). *Curr. Opin. Chem. Biol.* 15 475–481. 10.1016/j.cbpa.2011.05.024 21684802

[B151] Muegge I. (2006). PMF scoring revisited. *J. Med. Chem.* 49 5895–5902. 10.1021/jm050038s 17004705

[B152] Mullard A. (2014). New drugs cost US$2.6 billion to develop. *Nat. Rev. Drug Discov.* 13 877–877. 10.1038/nrd4507

[B153] Murphy R. B. Repasky M. P. Greenwood J. R. Tubert-Brohman I. Jerome S. Annabhimoju R. (2016). WScore: a flexible and accurate treatment of explicit water molecules in ligand-receptor docking. *J. Med. Chem.* 59 4364–4384. 10.1021/acs.jmedchem.6b00131 27054459

[B154] Mysinger M. M. Carchia M. Irwin J. J. Shoichet B. K. (2012). Directory of useful decoys, enhanced (DUD-E): better ligands and decoys for better benchmarking. *J. Med. Chem.* 55 6582–6594. 10.1021/jm300687e 22716043 PMC3405771

[B155] Nguyen D. D. Cang Z. Wu K. Wang M. Cao Y. Wei G.-W. (2018). Mathematical deep learning for pose and binding affinity prediction and ranking in D3R grand challenges. *J. Comput. Aided Mol. Des.* [Epub ahead of print]. 30116918 10.1007/s10822-018-0146-6 PMC7163798

[B156] Numao S. Damager I. Li C. Wrodnigg T. M. Begum A. Overall C. M. (2004). In situ extension as an approach for identifying novel α-amylase inhibitors. *J. Biol. Chem.* 279 48282–48291. 10.1074/jbc.M406804200 15304511

[B157] Nunes-Alves A. Arantes G. M. (2014). Ligand-receptor affinities computed by an adapted linear interaction model for continuum electrostatics and by protein conformational averaging. *J. Chem. Inform. Model.* 54 2309–2319. 10.1021/ci500301s 25076043

[B158] Ouyang X. Zhou S. Su C. T. T. Ge Z. Li R. Kwoh C. K. (2013). Covalent dock: automated covalent docking with parameterized covalent linkage energy estimation and molecular geometry constraints. *J. Comput. Chem.* 34 326–336. 10.1002/jcc.23136 23034731

[B159] Pagadala N. S. Syed K. Tuszynski J. (2017). Software for molecular docking: a review. *Biophys. Rev.* 9 91–102. 10.1007/s12551-016-0247-1 28510083 PMC5425816

[B160] Park M.-S. Dessal A. L. Smrcka A. V. Stern H. A. (2009). Evaluating docking methods for prediction of binding affinities of small molecules to the g protein βγ subunits. *J. Chem. Inform. Model.* 49 437–443. 10.1021/ci800384q 19434844 PMC2846521

[B161] Park S.-J. Kufareva I. Abagyan R. (2010). Improved docking, screening and selectivity prediction for small molecule nuclear receptor modulators using conformational ensembles. *J. Comput. Aided Mol. Des.* 24 459–471. 10.1007/s10822-010-9362-4 20455005 PMC2881208

[B162] Pason L. P. Sotriffer C. A. (2016). Empirical scoring functions for affinity prediction of protein-ligand complexes. *Mol. Inform.* 35 541–548. 10.1002/minf.201600048 27870243

[B163] Paulsen J. L. Anderson A. C. (2009). Scoring ensembles of docked protein: ligand interactions for virtual lead optimization. *J. Chem. Inform. Model.* 49:2813. 10.1021/ci9003078 19950979 PMC2819002

[B164] Pecina A. Brynda J. Vrzal L. Gnanasekaran R. Hořejší M. Eyrilmez S. M. (2018). Ranking power of the SQM/COSMO scoring function on carbonic anhydrase II-inhibitor complexes. *ChemPhysChem* 19 873–879. 10.1002/cphc.201701104 29316128

[B165] Pereira J. C. Caffarena E. R. dos Santos C. N. (2016). Boosting docking-based virtual screening with deep learning. *J. Chem. Inform. Model.* 56 2495–2506. 10.1021/acs.jcim.6b00355 28024405

[B166] Petukh M. Stefl S. Alexov E. (2013). The role of protonation states in ligand-receptor recognition and binding. *Curr. Pharm. Des.* 19 4182–4190. 23170880 10.2174/1381612811319230004 PMC3625499

[B167] Pierce A. C. Sandretto K. L. Bemis G. W. (2002). Kinase inhibitors and the case for CH...O hydrogen bonds in protein-ligand binding. *Proteins* 49 567–576. 10.1002/prot.10259 12402365

[B168] Pires D. E. V. Ascher D. B. (2016). CSM-lig: a web server for assessing and comparing protein–small molecule affinities. *Nucleic Acids Res.* 44 W557–W561. 10.1093/nar/gkw390 27151202 PMC4987933

[B169] Poornima C. S. Dean P. M. (1995). Hydration in drug design. 1. Multiple hydrogen-bonding features of water molecules in mediating protein-ligand interactions. *J. Comput. Aided Mol. Des.* 9 500–512. 8789192 10.1007/BF00124321

[B170] Qiu D. Shenkin P. S. Hollinger F. P. Still W. C. (1997). The GB/SA continuum model for solvation. A fast analytical method for the calculation of approximate born radii. *J. Phys. Chem. A* 101 3005–3014. 10.1021/jp961992r

[B171] Ragoza M. Hochuli J. Idrobo E. Sunseri J. Koes D. R. (2017). Protein-ligand scoring with convolutional neural networks. *J. Chem. Inform. Model.* 57 942–957. 10.1021/acs.jcim.6b00740 28368587 PMC5479431

[B172] Raha K. Merz K. M. (2005). Large-scale validation of a quantum mechanics based scoring function: predicting the binding affinity and the binding mode of a diverse set of protein-ligand complexes. *J. Med. Chem.* 48 4558–4575. 10.1021/jm048973n 15999994

[B173] Rarey M. Kramer B. Lengauer T. Klebe G. (1996). A fast flexible docking method using an incremental construction algorithm. *J. Mol. Biol.* 261 470–489. 10.1006/jmbi.1996.0477 8780787

[B174] Ravindranath P. A. Forli S. Goodsell D. S. Olson A. J. Sanner M. F. (2015). AutoDockFR: advances in protein-ligand docking with explicitly specified binding site flexibility. *PLoS Comput. Biol.* 11:e1004586. 10.1371/journal.pcbi.1004586 26629955 PMC4667975

[B175] Réau M. Langenfeld F. Zagury J.-F. Lagarde N. Montes M. (2018). Decoys selection in benchmarking datasets: overview and perspectives. *Front. Pharmacol.* 9:11. 10.3389/fphar.2018.00011 29416509 PMC5787549

[B176] Riniker S. Barandun L. J. Diederich F. Krämer O. Steffen A. van Gunsteren W. F. (2012). Free enthalpies of replacing water molecules in protein binding pockets. *J. Comput. Aided Mol. Des.* 26 1293–1309. 10.1007/s10822-012-9620-8 23247390

[B177] Rognan D. (2017). The impact of in silico screening in the discovery of novel and safer drug candidates. *Pharmacol. Ther.* 175 47–66. 10.1016/j.pharmthera.2017.02.034 28223231

[B178] Rognan D. Lauemoller S. L. Holm A. Buus S. Tschinke V. (1999). Predicting binding affinities of protein ligands from three-dimensional models: application to peptide binding to class I major histocompatibility proteins. *J. Med. Chem.* 42 4650–4658. 10579827 10.1021/jm9910775

[B179] Ryde U. Söderhjelm P. (2016). Ligand-binding affinity estimates supported by quantum-mechanical methods. *Chem. Rev.* 116 5520–5566. 10.1021/acs.chemrev.5b00630 27077817

[B180] Santos-Martins D. (2016). Interaction with specific HSP90 residues as a scoring function: validation in the D3R Grand Challenge 2015. *J. Comput. Aided Mol. Des.* 30 731–742. 10.1007/s10822-016-9943-y 27549813

[B181] Santos-Martins D. Forli S. Ramos M. J. Olson A. J. (2014). AutoDock4Zn: an improved autodock force field for small-molecule docking to zinc metalloproteins. *J. Chem. Inform. Model.* 54 2371–2379. 10.1021/ci500209e 24931227 PMC4144784

[B182] Sastry G. M. Adzhigirey M. Day T. Annabhimoju R. Sherman W. (2013). Protein and ligand preparation: parameters, protocols, and influence on virtual screening enrichments. *J. Comput. Aided Mol. Des.* 27 221–234. 10.1007/s10822-013-9644-8 23579614

[B183] Schäfer H. Smith L. J. Mark A. E. van Gunsteren W. F. (2002). Entropy calculations on the molten globule state of a protein: side-chain entropies of α-lactalbumin. *Proteins Struct. Funct. Bioinform.* 46 215–224. 10.1002/prot.1166 11807950

[B184] Schneider G. Fechner U. (2005). Computer-based de novo design of drug-like molecules. *Nat. Rev. Drug Discov.* 4 649–663. 10.1038/nrd1799 16056391

[B185] Scholz C. Knorr S. Hamacher K. Schmidt B. (2015). DOCKTITE-a highly versatile step-by-step workflow for covalent docking and virtual screening in the molecular operating environment. *J. Chem. Inform. Model.* 55 398–406. 10.1021/ci500681r 25541749

[B186] Seifert M. H. J. (2009). Targeted scoring functions for virtual screening. *Drug Discov. Today* 14 562–569. 10.1016/j.drudis.2009.03.013 19508918

[B187] Shao J. (1993). Linear model selection by cross-validation. *J. Am. Stat. Assoc.* 88 486–494. 10.2307/2290328

[B188] Shoichet B. K. (2006). Interpreting steep dose-response curves in early inhibitor discovery. *J. Med. Chem.* 49 7274–7277. 10.1021/jm061103g 17149857

[B189] Sitkoff D. Sharp K. A. Honig B. (1994). Accurate calculation of hydration free energies using macroscopic solvent models. *J. Phys. Chem.* 98 1978–1988. 10.1021/j100058a043

[B190] Smith R. D. Damm-Ganamet K. L. Dunbar J. B. Ahmed A. Chinnaswamy K. Delproposto J. E. (2016). CSAR benchmark exercise 2013: evaluation of results from a combined computational protein design, docking, and scoring/ranking challenge. *J. Chem. Inform. Model.* 56 1022–1031. 10.1021/acs.jcim.5b00387 26419257 PMC6588165

[B191] Sotriffer C. (2012). “Scoring functions for protein-ligand interactions,” in *Protein-Ligand Interactions*, ed. Gohlke H. (Weinheim: Wiley-VCH Verlag GmbH & Co. KGaA), 237–263.

[B192] Sotriffer C. Matter H. (2011). “The challenge of affinity prediction: scoring functions for structure-based virtual screening,” in *Methods and Principles in Medicinal Chemistry*, ed. Sotriffer C. (Weinheim: Wiley-VCH Verlag GmbH & Co. KGaA), 177–221.

[B193] Sotriffer C. A. Sanschagrin P. Matter H. Klebe G. (2008). SFCscore: scoring functions for affinity prediction of protein-ligand complexes. *Proteins* 73 395–419. 10.1002/prot.22058 18442132

[B194] Spiliotopoulos D. Kastritis P. L. Melquiond A. S. J. Bonvin A. M. J. J. Musco G. Rocchia W. (2016). dMM-PBSA: a new HADDOCK scoring function for protein-peptide docking. *Front. Mol. Biosci.* 3:46. 10.3389/fmolb.2016.00046 27630991 PMC5006095

[B195] Spyrakis F. Cavasotto C. N. (2015). Open challenges in structure-based virtual screening: receptor modeling, target flexibility consideration and active site water molecules description. *Arch. Biochem. Biophys.* 583 105–119. 10.1016/j.abb.2015.08.002 26271444

[B196] Still W. C. Tempczyk A. Hawley R. C. Hendrickson T. (1990). Semianalytical treatment of solvation for molecular mechanics and dynamics. *J. Am. Chem. Soc.* 112 6127–6129. 10.1021/ja00172a038

[B197] Stouch T. (2008). Editorial: special issue on “evaluation of computational methods.” *J. Comput. Aided Mol. Des.* 22:131. 10.1007/s10822-008-9197-4 18338227

[B198] Sun H. Li Y. Tian S. Xu L. Hou T. (2014). Assessing the performance of MM/PBSA and MM/GBSA methods. 4. Accuracies of MM/PBSA and MM/GBSA methodologies evaluated by various simulation protocols using PDBbind data set. *Phys. Chem. Chem. Phys.* 16 16719–16729. 10.1039/c4cp01388c 24999761

[B199] Tanford C. (1980). *The Hydrophobic Effect: Formation of Micelles and Biological Membranes*, 2nd Edn. New York, NY: Wiley.

[B200] Teramoto R. Fukunishi H. (2007). Supervised consensus scoring for docking and virtual screening. *J. Chem. Inform. Model.* 47 526–534. 10.1021/ci6004993 17295466

[B201] Terp G. E. Johansen B. N. Christensen I. T. Jørgensen F. S. (2001). A new concept for multidimensional selection of ligand conformations (multiselect) and multidimensional scoring (multiscore) of protein-ligand binding affinities. *J. Med. Chem.* 44 2333–2343. 10.1021/jm001090l 11428927

[B202] Totrov M. Abagyan R. (1997). Flexible protein-ligand docking by global energy optimization in internal coordinates. *Proteins Suppl.* 1 215–220. 9485515 10.1002/(sici)1097-0134(1997)1+<215::aid-prot29>3.3.co;2-i

[B203] Totrov M. Abagyan R. (1999). *Derivation of Sensitive Discrimination Potential for Virtual Ligand Screening.* New York, NY: ACM Press, 312–320. 10.1145/299432.299509

[B204] Trani J. M. D. Cesco S. D. O’Leary R. Plescia J. Nascimento C. J. do Moitessier N. (2018). Rapid measurement of inhibitor binding kinetics by isothermal titration calorimetry. *Nat. Commun.* 9:893. 10.1038/s41467-018-03263-3 29497037 PMC5832847

[B205] Trott O. Olson A. J. (2010). AutoDock vina: improving the speed and accuracy of docking with a new scoring function, efficient optimization and multithreading. *J. Comput. Chem.* 31 455–461. 10.1002/jcc.21334 19499576 PMC3041641

[B206] Tuffery P. Derreumaux P. (2012). Flexibility and binding affinity in protein-ligand, protein-protein and multi-component protein interactions: limitations of current computational approaches. *J. R. Soc. Interface* 9 20–33. 10.1098/rsif.2011.0584 21993006 PMC3223636

[B207] Tuley A. Fast W. (2018). The taxonomy of covalent inhibitors. *Biochemistry (Mosc.)* 57 3326–3337. 10.1021/acs.biochem.8b00315 29689165 PMC6016374

[B208] Usha T. Shanmugarajan D. Goyal A. K. Kumar C. S. Middha S. K. (2017). Recent updates on computer-aided drug discovery: time for a paradigm shift. *Curr. Top. Med. Chem.* 17 3296–3307. 10.2174/1568026618666180101163651 29295698

[B209] van Zundert G. C. P. Rodrigues J. P. G. L. M. Trellet M. Schmitz C. Kastritis P. L. Karaca E. (2016). The HADDOCK2.2 web server: user-friendly integrative modeling of biomolecular complexes. *J. Mol. Biol.* 428 720–725. 10.1016/j.jmb.2015.09.014 26410586

[B210] Velec H. F. G. Gohlke H. Klebe G. (2005). DrugScore(CSD)-knowledge-based scoring function derived from small molecule crystal data with superior recognition rate of near-native ligand poses and better affinity prediction. *J. Med. Chem.* 48 6296–6303. 10.1021/jm050436v 16190756

[B211] Verdonk M. L. Berdini V. Hartshorn M. J. Mooij W. T. M. Murray C. W. Taylor R. D. (2004). Virtual screening using protein-ligand docking: avoiding artificial enrichment. *J. Chem. Inform. Model.* 44 793–806. 10.1021/ci034289q 15154744

[B212] Verdonk M. L. Chessari G. Cole J. C. Hartshorn M. J. Murray C. W. Nissink J. W. M. (2005). Modeling water molecules in protein-ligand docking using GOLD. *J. Med. Chem.* 48 6504–6515. 10.1021/jm050543p 16190776

[B213] Villoutreix B. Eudes R. Miteva M. (2009). Structure-based virtual ligand screening: recent success stories. *Comb. Chem. High Throughput Screen.* 12 1000–1016. 10.2174/138620709789824682 20025565

[B214] Vogel S. M. Bauer M. R. Boeckler F. M. (2011). DEKOIS: demanding evaluation kits for objective in silico screening – A versatile tool for benchmarking docking programs and scoring functions. *J. Chem. Inf. Model.* 51 2650–2665. 10.1021/ci2001549 21774552

[B215] Wallach I. Dzamba M. Heifets A. (2015). AtomNet: a deep convolutional neural network for bioactivity prediction in structure-based drug discovery. arXiv:1510.02855 [Preprint].

[B216] Wang C. Zhang Y. (2017). Improving scoring-docking-screening powers of protein–ligand scoring functions using random forest. *J. Comput. Chem.* 38 169–177. 10.1002/jcc.24667 27859414 PMC5140681

[B217] Wang J.-C. Lin J.-H. Chen C.-M. Perryman A. L. Olson A. J. (2011). Robust scoring functions for protein-ligand interactions with quantum chemical charge models. *J. Chem. Inform. Model.* 51 2528–2537. 10.1021/ci200220v 21932857 PMC4639406

[B218] Wang R. Lai L. Wang S. (2002). Further development and validation of empirical scoring functions for structure-based binding affinity prediction. *J. Comput. Aided Mol. Des.* 16 11–26. 12197663 10.1023/a:1016357811882

[B219] Wang R. Liu L. Lai L. Tang Y. (1998). SCORE: a new empirical method for estimating the binding affinity of a protein-ligand complex. *J. Mol. Model.* 4 379–394. 10.1007/s008940050096

[B220] Wang R. Lu Y. Wang S. (2003). Comparative evaluation of 11 scoring functions for molecular docking. *J. Med. Chem.* 46 2287–2303. 10.1021/jm0203783 12773034

[B221] Wang R. Wang S. (2001). How does consensus scoring work for virtual library screening? An idealized computer experiment. *J. Chem. Inform. Comput. Sci.* 41 1422–1426. 11604043 10.1021/ci010025x

[B222] Wang Y. Guo Y. Kuang Q. Pu X. Ji Y. Zhang Z. (2015). A comparative study of family-specific protein–ligand complex affinity prediction based on random forest approach. *J. Comput. Aided Mol. Des.* 29 349–360. 10.1007/s10822-014-9827-y 25527073

[B223] Wei B. Q. Baase W. A. Weaver L. H. Matthews B. W. Shoichet B. K. (2002). A model binding site for testing scoring functions in molecular docking. *J. Mol. Biol.* 322 339–355. 12217695 10.1016/s0022-2836(02)00777-5

[B224] Wei D. Zheng H. Su N. Deng M. Lai L. (2010). Binding energy landscape analysis helps to discriminate true hits from high-scoring decoys in virtual screening. *J. Chem. Inform. Model.* 50 1855–1864. 10.1021/ci900463u 20968314

[B225] Williams D. H. Bardsley B. (1999). Estimating binding constants – The hydrophobic effect and cooperativity. *Perspect. Drug Discov. Des.* 17 43–59. 10.1023/A:1008770523049

[B226] Wójcikowski M. Ballester P. J. Siedlecki P. (2017). Performance of machine-learning scoring functions in structure-based virtual screening. *Sci. Rep.* 7:46710. 10.1038/srep46710 28440302 PMC5404222

[B227] Yang J.-M. Chen Y.-F. Shen T.-W. Kristal B. S. Hsu D. F. (2005). Consensus scoring criteria for improving enrichment in virtual screening. *J. Chem. Inform. Model.* 45 1134–1146. 10.1021/ci050034w 16045308

[B228] Yang Y. Lightstone F. C. Wong S. E. (2013). Approaches to efficiently estimate solvation and explicit water energetics in ligand binding: the use of WaterMap. *Exp. Opin. Drug Discov.* 8 277–287. 10.1517/17460441.2013.749853 23286874

[B229] Yang Z. Liu Y. Chen Z. Xu Z. Shi J. Chen K. (2015). A quantum mechanics-based halogen bonding scoring function for protein-ligand interactions. *J. Mol. Model.* 21:138. 10.1007/s00894-015-2681-6 25957658

[B230] Yilmazer N. D. Korth M. (2016). Prospects of applying enhanced semi-empirical QM methods for 2101 virtual drug design. *Curr. Med. Chem.* 23 2101–2111. 27183985 10.2174/0929867323666160517120005

[B231] Yuriev E. Holien J. Ramsland P. A. (2015). Improvements, trends, and new ideas in molecular docking: 2012-2013 in review: improvements, trends, and new ideas in molecular docking. *J. Mol. Recognit.* 28 581–604. 10.1002/jmr.2471 25808539

[B232] Yuriev E. Ramsland P. A. (2013). Latest developments in molecular docking: 2010-2011 in review. *J. Mol. Recognit. JMR* 26 215–239. 10.1002/jmr.2266 23526775

[B233] Zhang L. Tan J. Han D. Zhu H. (2017). From machine learning to deep learning: progress in machine intelligence for rational drug discovery. *Drug Discov. Today* 22 1680–1685. 10.1016/j.drudis.2017.08.010 28881183

[B234] Zhang X. Perez-Sanchez H. Lightstone F. C. (2017). A comprehensive docking and MM/GBSA rescoring study of ligand recognition upon binding antithrombin. *Curr. Top. Med. Chem.* 17 1631–1639. 10.2174/1568026616666161117112604 27852201 PMC5403970

[B235] Zheng Z. Merz K. M. (2011). Ligand identification scoring algorithm (LISA). *J. Chem. Inform. Model.* 51 1296–1306. 10.1021/ci2000665 21561101 PMC3124579

[B236] Zhu K. Borrelli K. W. Greenwood J. R. Day T. Abel R. Farid R. S. (2014). Docking covalent inhibitors: a parameter free approach to pose prediction and scoring. *J. Chem. Inform. Model.* 54 1932–1940. 10.1021/ci500118s 24916536

[B237] Zilian D. Sotriffer C. A. (2013). SFCscore RF: a random forest-based scoring function for improved affinity prediction of proteinligand complexes. *J. Chem. Inf. Model.* 53 1923–1933. 10.1021/ci400120b 23705795

[B238] Zimmermann M. O. Lange A. Boeckler F. M. (2015). Evaluating the potential of halogen bonding in molecular design: automated scaffold decoration using the new scoring function XBScore. *J. Chem. Inform. Model.* 55 687–699. 10.1021/ci5007118 25654403

[B239] Zou X. Kuntz I. D. (1999). Inclusion of solvation in ligand binding free energy calculations using the generalized-born model. *J. Am. Chem. Soc.* 121 8033–8043. 10.1021/ja984102p

